# Dissociation of Rpb4 from RNA polymerase II is important for yeast functionality

**DOI:** 10.1371/journal.pone.0206161

**Published:** 2018-10-25

**Authors:** Lea Duek, Oren Barkai, Ron Elran, Isra Adawi, Mordechai Choder

**Affiliations:** Department of Molecular Microbiology, Rappaport Faculty of Medicine, Technion–Israel Institute of Technology, Haifa, Israel; Medical Faculty Mannheim, University of Heidelberg, GERMANY

## Abstract

Rpb4 is an RNA polymerase II (Pol II) subunit that binds Pol II transcripts co-transcriptionally, accompanies them to the cytoplasm and modulates mRNA export, translation and decay by interacting with cytoplasmic RNA modulators. The importance of the cytoplasmic roles of Rpb4 was challenged by a study reporting that the phenotype of *rpb2Δ rpb4Δ* cells can be rescued by an Rpb2-Rpb4 fusion protein, assuming that its Rpb4 moiety cannot dissociate from Pol II and functions in the cytoplasm. Here we demonstrate that although the fusion protein supports normal transcription, it adversely affects mRNA decay, cell proliferation and adaptability–e.g., response to stress. These defects are similar, albeit milder, than the defects that characterize *rpb4*Δ cells. At least two mechanisms alleviate the deleterious effect of the fusion protein. First, a portion of this fusion protein is cleaved into free Rpb2 and Rpb4. The free Rpb4 is functional, as it binds mRNAs and polysomes, like WT Rpb4. Second, the fusion protein is also capable of binding poly(A)+ mRNAs in the cytoplasm, in an Rpb7-mediated manner, probably complementing the functions of the diminished Rpb4. Collectively, normal coupling between mRNA synthesis and decay requires wild-type configuration of Rpb4, and fusing Rpb4 to Rpb2 compromises this coupling.

## Introduction

Regulation of gene expression is mediated by distinct stages (e.g., transcription translation). Our lab investigates the linkage among the different stages. One focus of our work is two proteins, Rpb4 and Rpb7 that function as a heterodimer and participate in transcription by binding RNA polymerase II (Pol II) [1–3]. They mediate transcription initiation elongation and polyadenylation as well as dephosphorylation of the C-terminal domain of Rpb1 [3–16]. In addition, they stimulate mRNA export [17] translation [18,19] and decay [20–23], by binding mRNAs and key regulatory factors. More specifically, works from a number of labs have previously discovered that the Rpb4/7 heterodimer, or its archeal homologue, dissociates readily from Pol II [4,24], binds RNA [6,25,26] and mRNAs [18–20,27,28], primarily at their 3' untranslated regions [28] and associates with polysomes [18,19]. Rpb4 and Rpb7 bind components of the translation initiation complex 3 (eIF3), Nip1 and Hcr1 [18,19], bind components of the decay complex, Pat1, Lsm2, and Not5 and can be localized to processing bodies [18,19,21,22]. Rpb4 and Rpb7 are shuttling proteins. The export of Rpb4 from the nucleus to the cytoplasm is dependent on Pol II transcription, consistent with it being exported together with Pol II transcripts [29]. Importantly, we have shown that Rpb4/7 binds mRNA only in the context of Pol II and that its function in mRNA decay and translation depends on its interaction with Pol II [18,20], establishing a functional linkage between its different roles.

A previous publication by Schulz et al. [15] argued against the cytoplasmic role of Rpb4 in mRNA decay. Specifically, the researchers expressed a chimeric *RPB2-RPB4* gene in a yeast strain deleted for both *RPB2* and *RPB4*. Thus, in this strain, Rpb4 is covalently fused to the Pol II core subunit Rpb2, and the fusion protein is the sole source of Rpb2 and Rpb4. Introducing the fusion gene is a challenge that the cells have never confronted with. Investigators have previously reported that, in order to survive new and unforeseen challenges, yeast cells can solve the challenges by eliciting exploratory dynamical processes [30–32] (see Discussion). Schulz et al. found that the *Δrpb4* phenotype can be rescued by introducing the unnatural chimeric gene. This led them to conclude that Rpb4 functions exclusively in transcription, as no separation between Rpb4 and Pol II is possible due to the covalent linkage between Rpb4 and Rpb2. The underlying assumption of their work was that the fusion protein cannot function outside the context of Pol II.

We have further analyzed the strains that express the *RPB2-RPB4* chimeric gene as the sole source of Rpb2 and Rpb4 (named herein *RPB2-RPB4* strain) and found that, under optimal conditions, these strains proliferate slightly more slowly than wild-type. Moreover, under stress conditions, proliferation of these mutant cells becomes more severely compromised. Under some genetic background (*rpb7-29*), the chimeric gene becomes more deleterious. Interestingly, Schulz et al. reported that *RPB2-RPB4* mutant cells transcribe normally, but are defective in mRNA decay. However, because the defect of these cells in mRNA decay was substantially milder than the defect of *rpb4Δ* cells in mRNA decay, they proposed that it was not significant. We further analyzed the mRNA synthetic rates (SR) and decay rates (DRs) datasets, obtained by Schulz et al. [15]. Our analyses have corroborated the conclusion of Schulz et al. regarding transcription. Indeed, the fusion protein almost fully complements the function of Rpb2 and Rpb4 in transcription. Yet, when we evaluated the defect in mRNA decay, by determining the fold change of DRs in the mutant relative to WT, we found out that the relatively small changes in DRs in *RPB2-RPB4* mutant cells were correlated with the larger changes in DRs that characterize *rpb4Δ* cells. That is, mRNAs whose degradation rates were little dependent on Rpb4 were also little affected in *RPB2-RPB4* cells, whereas those that were highly dependent on Rpb4 were also more strongly affected by the fusion proteins. This correlation was found only between DRs of *RPB2-RPB4* and DRs of *rpb4Δ* strain and not when we performed similar analyses with other mutant strains carrying deletion in other mRNA decay factors. Based on our results, we propose that the defect of *RPB2-RPB4* cells in mRNA decay is significant and related to Rpb4 function. Thus, *RPB2-RPB4* strain transcribes normally but does not degrade mRNAs normally, demonstrating that transcription and mRNA decay are not necessarily coupled. We also found that a portion of the fusion protein is cleaved into free Rpb2 and free Rpb4. The free Rpb4 molecules are capable of binding mRNAs and polysomes, much like WT Rpb4. Unexpectedly, the full-length fusion protein binds mRNAs, via its Rpb4 moiety. These features of the fusion protein alleviate its adverse effect on DRs and permit almost normal cell proliferation under optimal conditions, but are insufficient to support efficient proliferation under stress and in mutants, such as in Rpb7-29 cells, in which the Rpb7-Rpb4 binding feature is compromised. Our results manifest, once again, the remarkable capacity of the cell to adapt to a new genetic disposition and illustrate the importance of normal configuration of Rpb4 for the coupling between mRNA synthesis and decay. Importantly, defective coupling affects cell phenotype mainly under non-optimal conditions, suggesting that this coupling is required mainly for proper responses to the environment.

## Results

### Expression of the *RPB2-RPB4* fusion gene slows down cell proliferation and compromises normal responses to stress

**Fig 1:**
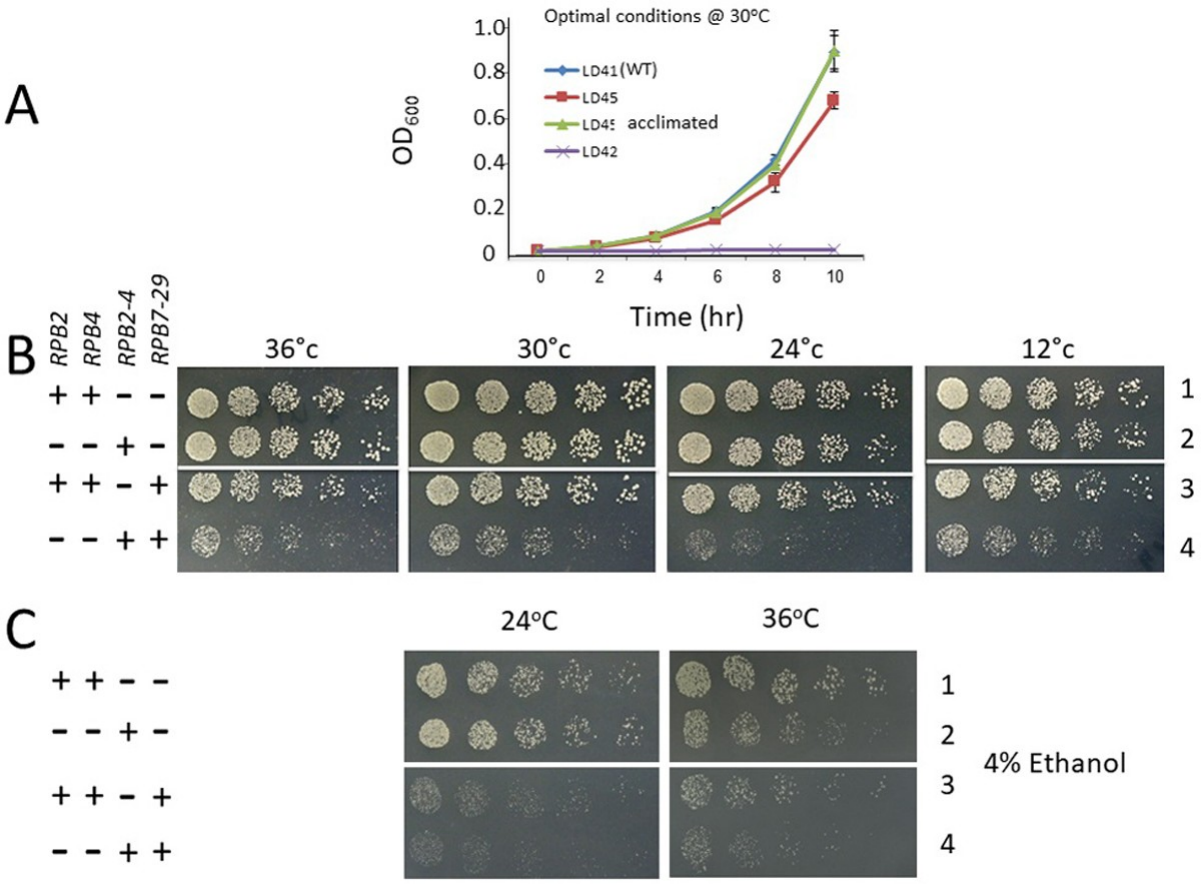
Expression of the *RPB2-RPB4* fusion gene compromises cell proliferation. **(A)** The yLD45 strain was grown in two parallel cultures in SC medium lacking leucine. One culture was supplemented with 10 μg/ml doxycycline (named "yLD45 acclimated") to repress expression of the Tet-off *RPB2*. After ~60 generations of growth in logarithmic phase (with intermittent dilutions), the two cultures were diluted into fresh medium containing doxycycline for 24 h (relatively short) acclimation to the drug. Cultures were then diluted, in triplicate, into fresh medium containing doxycycline and proliferation rate was measured. Data are presented as means ± SD (n = 3). yLD42 contains Tet-off-*RPB2* as the sole source of Rpb2, but otherwise is WT; yLD41 is a derivative of yLD42 that expresses *pRPB2*::*LEU2* and therefore proliferates as WT even in the presence of doxycycline. **(B)** Expression of *RPB2-RPB4* is deleterious in cells that overexpress *rpb7-29*. Spot test of 5-fold serial dilution of yMC798, yMC871, yMC923 and yMC924 (from top to bottom) grown on selective plates at the indicated temperatures. The first spot of each strain was of 5000 cells. **(C)** Spot test of 5-fold serial dilution of the indicated strains (same as in B) grown on selective plates containing 4% ethanol as the main carbon source. The first spot of each strain was of 5000 cells. The growth temperature is indicated above each panel.

Proliferation rate of cells expressing *RPB2-RPB4* fusion gene as the sole source of both Rpb2 and Rpb4 (named herein *RPB2-RPB4* cells) indicated that they proliferate like wild-type (WT) [15]. Freshly made *rpb4Δ* strain (but otherwise WT) proliferates slowly but "senior" *rpb4Δ* strain—having the same genetic background—proliferates faster. Thus, if allowed enough time, cells lacking *RPB4* adapt to the new genetic makeup and increase their proliferation rate [15]. We suspected that a similar acclimation might have occurred in the *RPB2-RPB4* strain. To minimize this acclimation, we introduced a plasmid encoding the *RPB2-RPB4* fusion gene into *rpb4*Δ cells that harbor Tet-off-*RPB2* in place of the natural *RPB2* (yLD45). In the absence of doxycycline, this strain co-expresses both Rpb2 (from the Tet-off-*RPB2*) and the fusion protein (S1A Fig). Upon the addition of doxycycline, the Tet-off *RPB2* is repressed (S1A Fig) and cells proliferation becomes dependent on the fusion gene. Cells that had never been exposed to doxycycline were cultured in doxycycline-containing medium, allowing relatively little time for acclimation, ~24h (this time of doxycycline treatment was required to obtain a full proliferation arrest of the control Tet-off *RPB2* cells [yLD42]). Under these conditions, the fusion protein was unable to fully restore the proliferation rate to that of WT cells (Fig 1A, compare “LD41” and “LD45”). We then allowed these cells to undergo ~ 60 cell divisions in the presence of doxycycline, when only the fusion protein provides the Rpb2 function, thus permitting cells to acclimate to the new genetic makeup. These "acclimated" cells proliferated similarly to WT cells (Fig 1A, compare “LD41” and “LD45 acclimated”). In contrast, cells that underwent the same number of cell divisions in the absence of doxycycline were not acclimated. This indicates that fusing Rpb2 to Rpb4 is partially deleterious only if free Rpb2 is absent. These results suggest that, when Rpb2 is expressed, the fusion protein provides the function of Rpb4. Although the deleterious effect of the fusion protein was not substantial (maybe owing to the gradual decrease of Rpb2 during the 24 h before the experiment begun), cells did respond to it and managed to reverse its effect on proliferation. The difference between the proliferation rate of “naïve” and “acclimated” cells was substantial enough, so that after ~60 cell generations, the "acclimated" cells took over the population and most cells in the population proliferated like WT.

We next examined whether *RPB2-RPB4* cells, which had the chance to acclimate during the creation of the strain and its handling, proliferate as WT. To this end, we mixed equal amount of *RPB2-RPB4* and WT cells, each carrying a different selectable marker, and co-cultured them. As shown in S1B Fig, the WT cells gradually took over the population indicating that *RPB2-RPB4* cells proliferated more slowly than WT. Thus, even "acclimated" *RPB2-RPB4* cells grow more slowly than WT.

Rpb4 functions in complex with Rpb7, which recruits Rpb4 to Pol II [1,3]. The Rpb7-29 mutant form is defective in binding Rpb4 [29]. We therefore checked whether this mutant also affects cell proliferation. WT cells overexpressing *rpb7-29* proliferated almost like WT cells (Fig 1B, compare the colony size in lane 1 and 3). In contrast, over-expressing *rpb7-29* in *RPB2-RPB4* cells affected their proliferation rate more severely, even under optimal conditions (Fig 1B, compare the number of colonies and their size in lane 3 and 4). These phenotypes suggest that *RPB2-RPB4* cells are more dependent on Rpb7 than WT.

Strains lacking *RPB4* do not cope well with stress, such as high temperatures and ethanol, [4,5,17,33–36]. We therefore evaluated the response of the *RPB2-RPB4* strain to 4% ethanol and high temperature (36°C). Under these conditions of stress, the *RPB2-RPB4* strain proliferated more slowly than WT cells (Fig 1C, 36°C compare colonies size in lane 1 and 2), indicating that the fusion protein is deleterious under severe stress. Over-expressing *rpb7-29* further compromised proliferation of WT cells (Fig 1C compare lane 1 and 3). Importantly, over-expressing *rpb7-29* compromised proliferation of *RPB2-RPB4* cells more than that of WT cells (Fig 1C compare lane 3 and 4).

Collectively, the results of Fig 1 demonstrate that the fusion gene adversely affects cell proliferation under all conditions tested. Under non-optimal conditions, e.g., in the presence of ethanol or extreme temperature (Fig 1B and 1C) or when raffinose is used as the main carbon source (results not shown), the deleterious effect of the fusion gene is more apparent. Finally, in some genetic makeups (i.e., *rpb7-29*), fusing Rpb4 to Rpb2 strongly affects cell proliferation, suggesting that the separation between Rpb4 and Rpb2 in these cells becomes critical for efficient proliferation and proper adaptation to the environment.

### *RPB2-RPB4* cells transcribe normally but do not degrade mRNAs normally

The deleterious effect of the fusion protein prompted us to investigate the effect of the fusion proteins on mRNA synthesis and decay. Schulz et al have determined the mRNA synthetic rates (SRs) and mRNA decay rates (DRs) of *RPB2-RPB4* cells, as well as *rpb4Δ* cells [15]. The median synthetic rates of *rpb4*Δ cells was 8-fold smaller than the WT. In contrast, the median synthetic rates of *RPB2-RPB4* cells was only 14% smaller than that of WT, and only 0.8% of genes exhibited rates that changed by more than twofold. Thus, the fusion protein can recover almost entirely the transcription defect of *rpb4Δ* cells. Indeed, we found that transcription rates of *RPB2-RPB4* cells is highly correlated with that of WT cells (r = 0.98) (results not shown).

**Fig 2:**
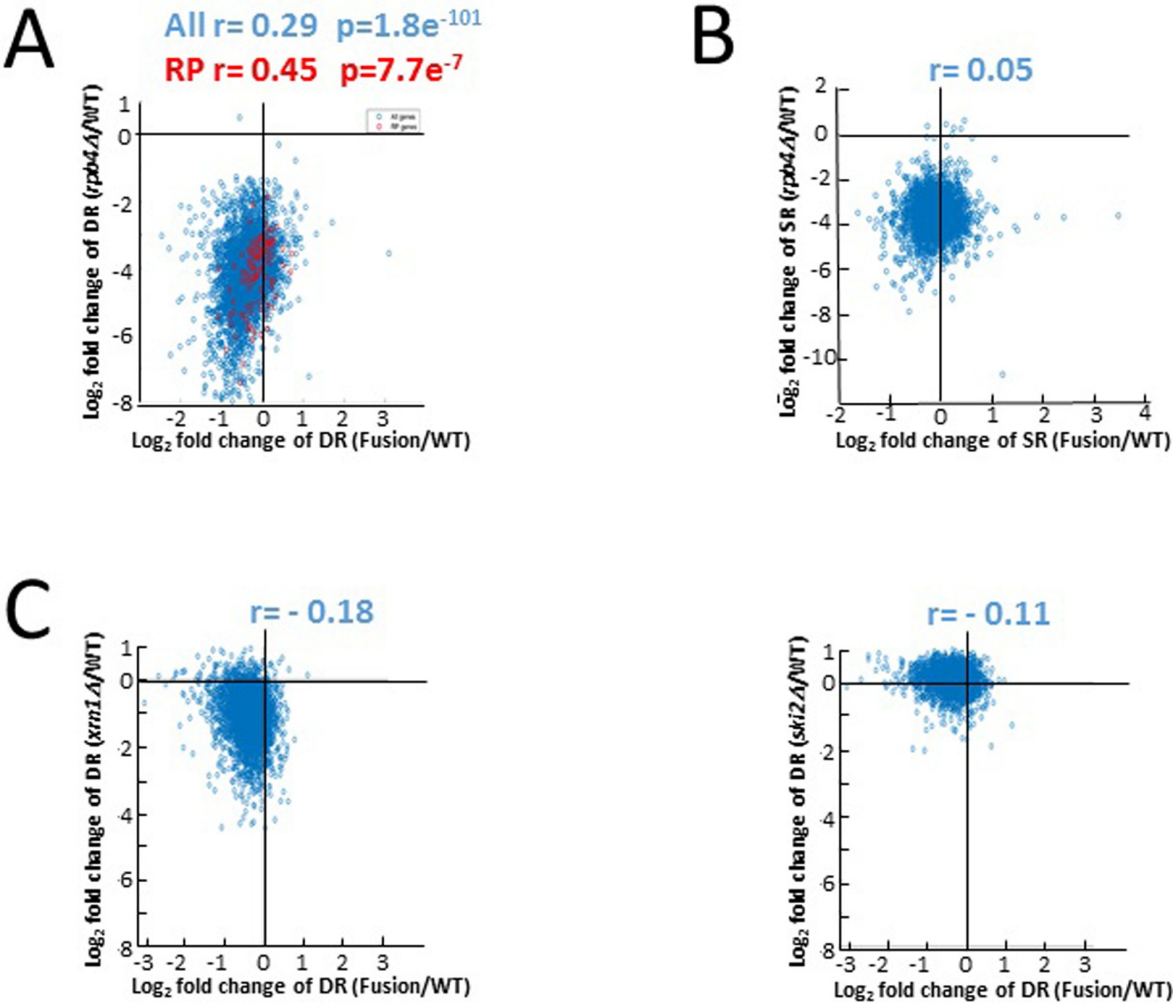
Correlation between mRNA synthesis and decay rates in *RPB2-RPB4* cells against those in *rpb4*Δ cells. mRNA synthesis rates (SRs) and decay rates (DRs) data of *rpb4*Δ and of *RPB2-RPB4* strains were taken from Schulz et al. [15]; DR data of the other strains were taken from [39]. **(A)** Fold changes in DRs for *rpb4*Δ cells (log_2_ folds of *rpb4*Δ/WT, y axis) against fold changes in DRs for *RPB2-RPB4* cells (log_2_ folds of *RPB2-RPB4*/WT, x axis). Each spot corresponds to one mRNA. Red spot represent mRNAs encoding ribosomal proteins whose decay rates are more dependent on Rpb4 than other mRNAs [21]. Spearman correlation was used to determine the correlation. **(B)** Correlation of SRs. Analysis similar to that in A was performed on SRs data. **(C)** Correlation analyses of DRs for *xrn1*Δ and *ski2*Δ strains against *RPB2-RPB4* strain were performed as in A. All strains are isogenic. See also S2 Fig.

If the effect that Rpb4 has on mRNA decay is only via its effect on transcription, as suggested [15], and the separation between Rpb2 and Rpb4 is not required for mRNA decay, we expect that mRNA decay rate in *RPB2-RPB4* cells would be similar WT. However, the median decay rates was 34% smaller than that of WT, while 13.7% of genes exhibited rates that changed by more than twofold (17-fold more than the changes observed when SRs were compared) [15]. To examine whether this decrease is related to Rpb4 function and to evaluate the significance of this decrease, we correlated the fold change of DR of *RPB2-RPB4* strain relative to WT against the fold change of DR of *rpb4Δ* relative to WT. As shown in Fig 2A, a modest, yet significant, correlation was observed (r = 0.29; p = 1.8e^-101^). Previously, we reported that the effect of Rpb4 on DR is stronger for mRNA encoding ribosomal proteins (RP) [20]. Indeed, the correlation for RP genes was better than for all genes (r = 0.45; p = 7.7e^-7^). This means that mRNAs whose degradation rates were little dependent on Rpb4 were also little affected in *RPB2-RPB4* cells, whereas those that were highly dependent on Rpb4 were also strongly affected by the fusion proteins. This correlation was specific to *rpb4*Δ strain, as no correlation or even an inverse (negative) and marginal correlation was observed when DRs of *RPB2-RPB4* strain were compared against DRs of strains lacking other mRNA decay factors (Figs 2C and S2). The results therefore suggest that the effect of fusing Rpb4 to Rpb2 on DRs is similar, albeit weaker, to the effect that *RPB4* deletion has on DRs. Taken together, we conclude that the 34% decrease in the average DR is related to the function of Rpb4 in stimulating mRNA decay. In contrast with DR, no correlation was observed when SR was similarly examined (r = 0.05) (Fig 2B), consistent with the full transcriptional recovery by the fusion protein.

### Cleavage of the Rpb2-Rpb4 fusion protein produces a pool of free Rpb2 and Rpb4 molecules

**Fig 3:**
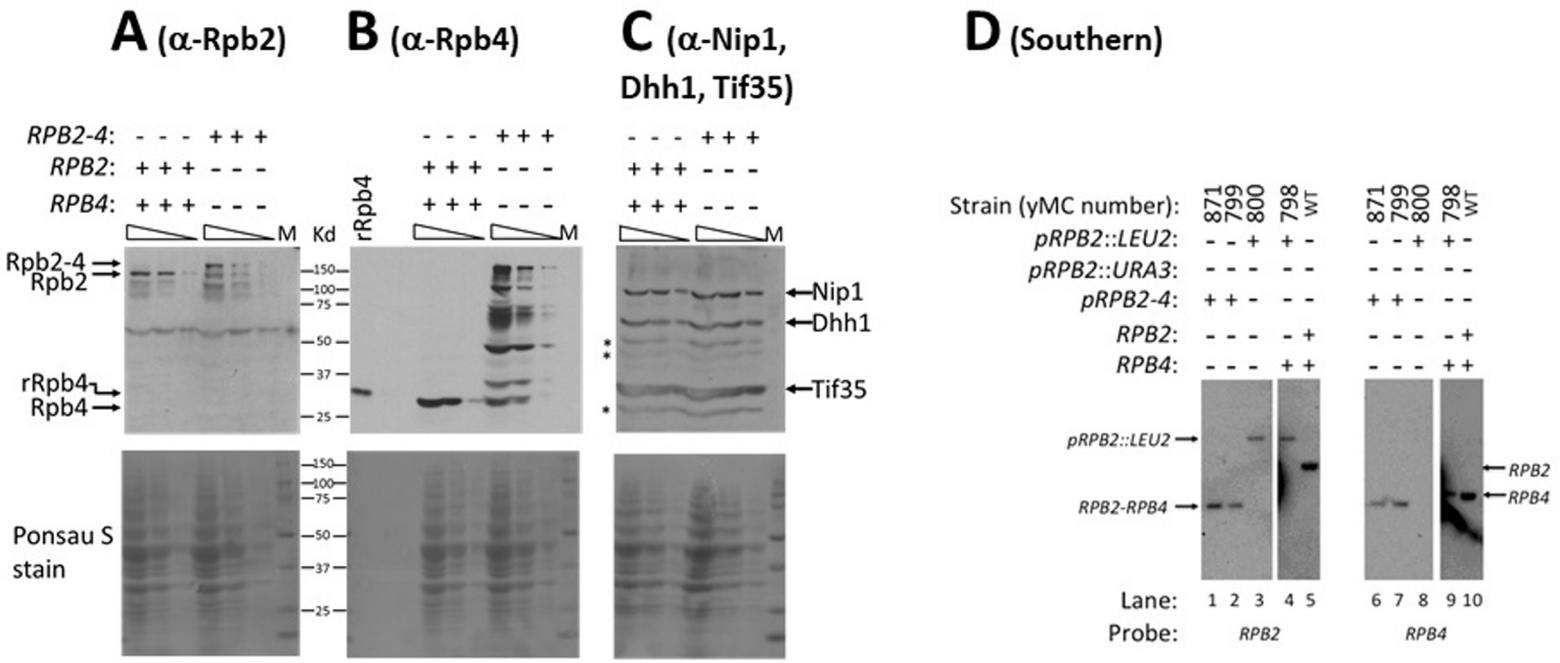
*RPB2-RPB4* cells do not contain other source of Rpb2 and Rpb4 and express Rpb2-Rbp4 fusion protein as well as Rpb2 and Rpb4 proteins. **(A-C)** Cell lysates of the indicated strains were loaded on polyacrylamide gel; each lysate was loaded in 3 lanes, containing either 200 μg, 70 μg or 20 μl of protein/lane. Three replicates, each containing 6 lanes, were thus loaded and analyzed by Western blotting assay; each membrane was reacted with either anti-Rpb2 Abs (A) or anti-Rpb4 Abs (B), or with a combination of anti-Nip1 + anti-Dhh1 + anti-Tif35 (C). Thirty ng of recombinant Rpb4-HISx6 (rRpb4), expressed in E. coli, was loaded in panel A to mark the position of Rpb4 (note that the rRpb4 migrated slower than Rpb4 due to an HISx6 tag that was used for its purification). Ponsau S stain of the membranes is shown underneath each respective panel. The pre-stained size marker (BioRad) is also shown. Asterisk indicates non-specific bands that characterize anti-Dhh1 and anti-Tif35 (results not shown). **(D)** DNA from the indicated strains was digested with Hind III, which cuts *RPB2* once at position 770 downstream to the translation start site (2930 bp upstream of the stop codon), and cuts *RPB4* ORF once at position 595 downstream to the translation start site (71 bp upstream of the stop codon). The DNA digest was subjected to Southern analysis, in duplicate, using either *RPB2* or *RPB4* probes as indicated. Y799 was constructed previously [15]. Y871 is an identical strain recreated in this work using the shuffling approach [15]. The 3535 bp Hind III fragment is indicated by an arrow (designated “*RPB2-RPB4*”). The position of the *RPB2*::*LEU2* plasmid and the endogenous *RPB2* and *RPB4* are also indicated. The DNA molecules of these strains were subject to PCR analyses, using various primer pairs. The results (not shown) were consistent with the results of this Southern experiment, indicating that the only source of Rpb2 and Rpb4 is a single *RPB2-RPB4* chimeric gene.

The deleterious effect of the fusion protein on cell proliferation and on mRNA decay is smaller than the effect of a deletion of *RPB4*. This prompted us to examine what might alleviate this adverse effect. We extracted proteins from *RPB2-RPB4* cells and analyzed them by Western blot assay, using either anti-Rpb2 or anti-Rpb4. As expected, the fusion protein was detected by both Rpb2 and Rpb4 antibodies (Fig 3A and 3B). However, we also detected degradation intermediates and free Rpb2 and Rpb4 proteins in the extract (Fig 3B). Free Rpb4 molecules were detected by various protein extraction methods, including extraction by 20% TCA or unusually high levels of denaturing reagents (48% Urea + 7% SDS) (S3 Fig). Analyzing several replicates using the TotalLab Quant software, we estimated the free Rpb4 to represent 10% ± 4% of the sum of the fusion protein and its degradation products. Comparing our results with those published previously [15] is compromised due to the different anti-Rpb4 antibodies used by the two groups. Importantly, transcription of the fusion gene is controlled by *RPB2* promoter and its translation is regulated by *RPB2* 5’ untranslated region [15]. Consequently, expression of the fusion protein is different than that of WT *RPB4*. Indeed, 6 independent western blot analyses using anti-Rpb4 Abs to detect both Rpb4 and the fusion proteins (including the degradation intermediates) we detected 2.65 ± 0.70 -fold higher Rpb2-Rpb4 molecules than that of Rpb4 molecules in WT cells. The level of the fusion protein is also higher than that of Rpb2 in WT cells (S1A Fig, compare the level of Rpb2 in WT cells to that of the fusion protein in yLD45). Importantly, although the free Rpb4 in *RPB2-RPB4* cells represent only ~10% of the fusion protein, it actually represents ~ 26% of the Rpb4 level in WT cells.

Existence of the free Rpb4 can result from genetic uncoupling between the two chimeric open reading frames due to a DNA recombination event, or post-translational cleavage of the fusion protein. To differentiate between these two possibilities, we performed Southern analysis using *RPB2* and *RPB4* probes. A Hind III fragment of the chimeric gene was readily detected by both *RPB2* and *RPB4* probes. Except for the band corresponding to the chimeric gene, this analysis detected no other sequences that could hybridize to *RPB2* or *RPB4* (Fig 3D, lanes 1, 2, 6 and 7). We recreated a strain carrying the chimeric gene as the sole source of *RPB2* and *RPB4* (yMC871) as described previously [15]. Analysis of the resulting strain produced identical results (Fig 3D, compare lanes 1 and 6 with 2 and 7, respectively). These results rule out the possibility that free Rpb2 and Rpb4 are encoded by independent genes, and are consistent with the post-translational cleavage option.

To determine whether cells can tolerate low level of Rpb4, we created a cDNA from *RPB4* mRNA and expressed it under the control of *GAL1* promoter, which is active when cells are cultured on galactose and repressed in response to glucose, and expressed it in cells lacking the natural *RPB4*. As shown in S4 Fig, the amount of Rpb4 in the glucose treated cells was 10-fold smaller than that in cells proliferated on galactose. The level was 54% that of the natural Rpb4 level in WT cells. It was not possible to obtain stronger repression, probably because the level of Rpb4 is also regulated at the level of translation. *RPB4* is essential for cell viability under temperature stress [[1,3]]. Significantly, these cells could proliferate on glucose at 37°C like WT, indicating that cells that produce only about one half of the normal Rpb4 level can proliferate like WT. Yet, it was higher than the free Rpb4 (~ 26%). We propose that production of free Rpb4 in *RPB2-RPB4* partially accounts for the mRNA decay capacity of these cells, which is better than in *rpb4Δ* cells, but not quite as WT (Fig 2).

### Defective polysomal profile obtained from *RPB2-RPB4* cells extract

**Fig 4:**
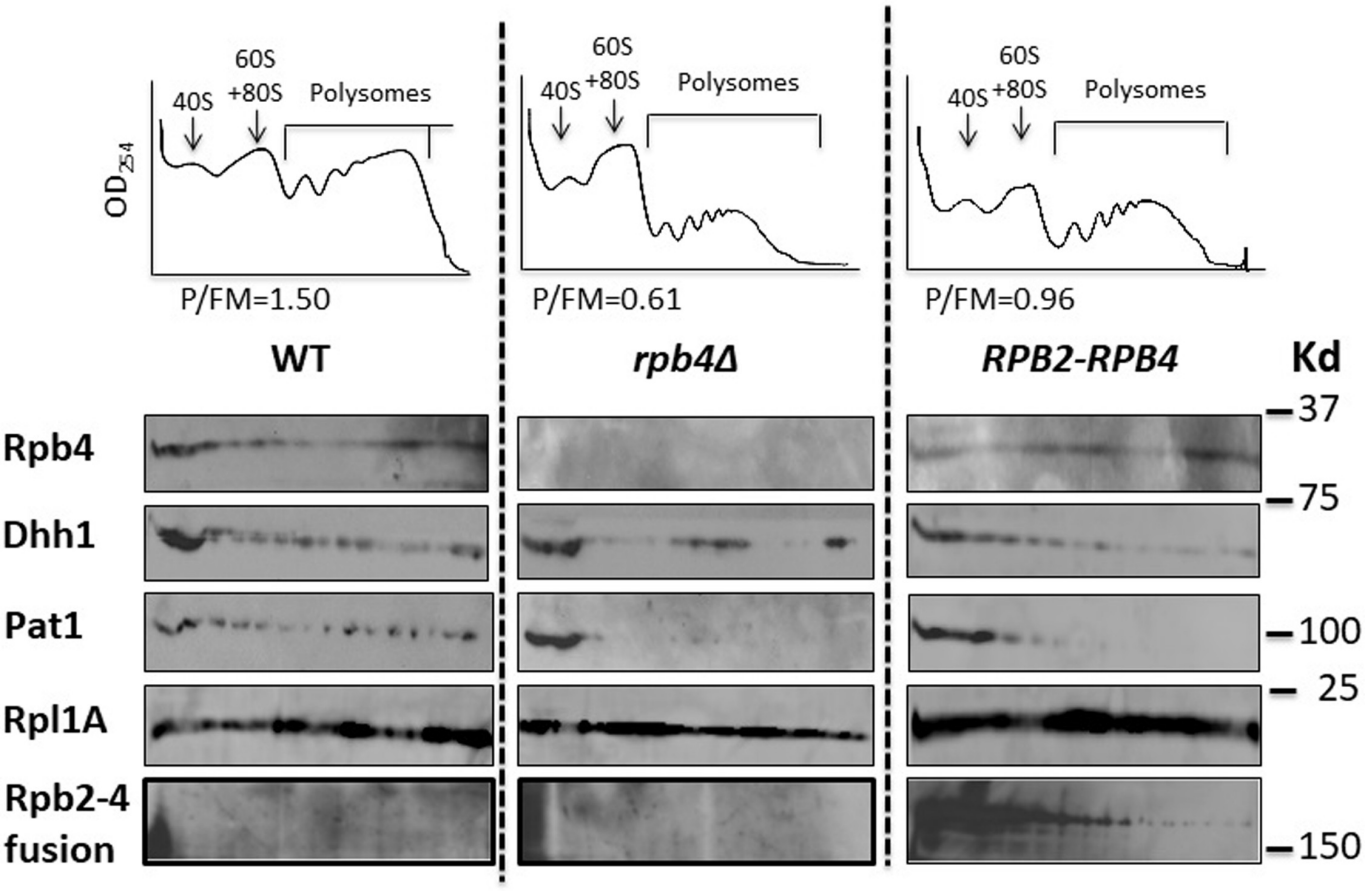
Association of the Rpb2-Rpb4 fusion protein and free Rpb4 with polysomes. Extracts from the indicated strains were subjected to polysomal fractionation as indicated in the Materials and methods [18]. Upper panel: the polysomal profiles for each strain. The ratio between polysomal RNA and free RNA + monosomal RNA (designated P/FM) is depicted below each profile [18]. Lower panel: The fractions were analyzed by Western blotting, using the indicated antibodies, to detect the polysomal marker Rpl1A, the translation factors Dhh1 (Dhh1 pattern is disrupted in some lanes due to inefficient blotting) and Pat1 as well as free Rpb4 and Rpb2-Rpb4 fusion proteins. Both the free Rpb4 and the fusion protein were detected by anti-Rpb4 Abs. To better detect the free Rpb4, twice as much protein was loaded per lane of the *RPB2-RPB4* sample.

Previously, Rpb4 was demonstrated to bind mRNAs and polysomes, thus regulating mRNA translation and decay (see Introduction). To determine the effect of the fusion protein on the polysomal profile, we performed polysomal fractionation analysis of WT, *rpb4*Δ and *RPB2-RPB4* cell extracts. As reported previously [18], the polysomal profile of *rpb4*Δ cells is defective, as reflected by the relatively low ratio between polysomal (representing translationally active ribosomes) to subpolysomal (non active 40S and 60S ribosomal subunits and 80S monosome) peaks (“P/FM”, indicated in the upper panels of Fig 4). The polysomal profile of the *RPB2*-*RPB4* strain reflects an intermediate state between WT and *rpb4*Δ (Fig 4; note that the P/FM value is between that of WT and *rpb*Δ). In addition, we analyzed the polysomal fractions by Western analysis. As shown in the autoradiogams of Fig 4, free Rpb4 was detected in the polysomal fractions derived from the *RPB2*-*RPB4* strain, similar to WT [[18,19], suggesting that the free Rpb4 functions like WT Rpb4. Interestingly, a small fraction of the fusion protein co-sedimented with the polysomes, consistent with binding of Rpb2-Rpb4 fusion protein with mRNAs (see next section). Pat1 and Dhh1 are translation factors that bind polysomes [37] (Fig 4). In the absence of *RPB4*, Pat1 sedimented mainly in the subpolysomal fractions (Fig 4, Pat1). This result suggests that Rpb4, a Pat1-binding protein [18], stabilizes the interaction of Pat1 with polysomes. The presence of Rpb2-Rpb4, and/or the free Rpb4, only partially restored Pat1 association with polysomes. Taken together, cells that express the fusion gene as the sole source of Rpb2 and Rpb4 give rise to defective polysomal profile; this defect is not as strong as that of *rpb4*Δ strain, probably due to the free Rpb4 and the small fraction of the fusion protein that co-sediments with the polysomes that together provide the function of Rpb4 in translation (see Discussion).

### The Rpb2-Rpb4 fusion protein binds mRNAs in an Rpb7-dependent manner

**Fig 5:**
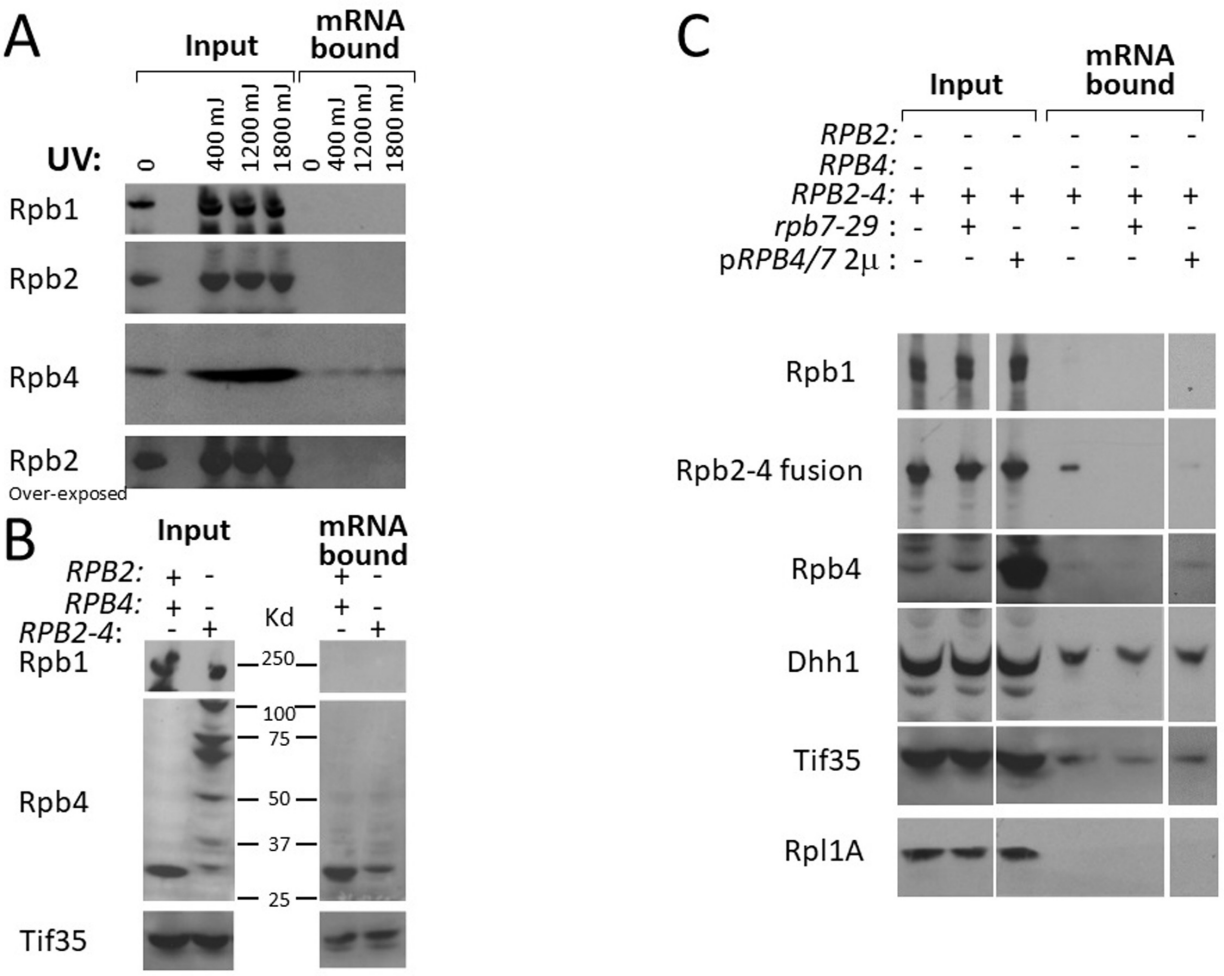
Endogenous Rpb4, Rpb2-Rpb4 fusion protein and the fusion-derived Rpb4 bind poly(A)+ RNAs. (A) Rpb4, but not Rpb2, binds poly(A)+ RNA. Live WT cells were irradiated with increasing doses of UV radiation, as indicated. RNPs were extracted from equal amount of cells and the poly(A)+ RNA was purified [27]. Proteins, which were captured by equal amount of mRNA (see Materials and methods), were analyzed by Western blotting. The membrane was cut at ~ 100 Kd into two pieces; the high MW portion was reacted with anti-Rpb2, whereas the low MW portion reacted with anti-Rpb4 antibodies. The high MW portion was then reacted with anti-Rpb1 Abs (anti-CTD). A 5-fold overexposure of the Rpb2 signal is shown in the lower panel. **(B)** Rpb4 derived from cleavage of the Rpb2-Rpb4 fusion protein binds poly(A)+ RNA. WT and *RPB2*-*RPB4* cells were irradiated with 1200 mJ of UV. Poly(A)+ RNA was purified under denaturing conditions and RNA-associated proteins were analyzed as in (A). The membrane was cut as in A and reacted with the either anti-Rpb1 or anti-Rpb4 Abs. The membrane piece with the low MW proteins was later reacted with anti-Tif35 Abs. Tif35 is an eIF3 component and is shown as a loading control. **(C)** The Rpb2-Rpb4 fusion protein binds poly(A)+ RNAs in an Rpb4/7-dependent manner. The *RPB2*-*RPB4* strain was transformed with a high-copy plasmid encoding an Rpb7 mutant defective in Rpb4-binding (Rpb7-29) or a high-copy plasmid carrying both *RPB*4 and *RPB7* ORFs including their respective 5’ and 3’ non-coding regions (p*RPB4/7*). Poly(A)+ RNA was purified and RNA-associated proteins were analyzed as in (A).

A number of investigators have previously shown that Rpb4 binds RNA *in vitro* [6,26] and mRNAs *in vivo* [18–20,27,28], consistent with its role in mRNA export, translation and decay. Garrido-Godino et al. [27] have recently demonstrated that Rpb4 is cross-linked with mRNAs by UV irradiation. We used the same approach to test the capacity of Rpb4 to cross-link to mRNAs, comparing WT and *RPB2*-*RPB4* strain. To this end, WT and *RPB2*-*RPB4 live* cells were UV irradiated to cross-link proteins to RNA *in vivo*. The mRNPs were then affinity purified using oligo (dT)_25_ beads and the associated proteins were detected by Western blot analysis. In WT cells, endogenous Rpb4 bound poly(A)-containing mRNAs, in a UV-dose dependent manner. This corroborates previous data indicating that Rpb4 binds mRNAs *in vivo* [18, 20, 27]. In contrast, endogenous Rpb2 did not cross-link with mRNA at all (Fig 5A). Significantly, in *RPB2*-*RPB4* cells, free Rpb4, but not Rpb1, was capable of binding mRNA (Fig 5B). In contrast, the degradation products of Rpb2-Rpb4 fusion protein exhibited little binding capacity (Fig 5B), suggesting that a portion of Rpb2 that is fused to Rpb4 compromises the capacity of Rpb4 to bind mRNAs—probably due to conformational incompatibility or cellular miss-localization. These results, and co-sedimentation of free Rpb4 with polysomes (Fig 4), indicate that the free Rpb4 which is cleaved off the fusion protein, but not other degradation products of the fusion protein, maintains the capacity of the WT Rpb4 to bind mRNA.

Importantly, since the UV cross-linking occurred *in vivo*, our observation that the degradation products were not detected among the mRNA bound proteins, whereas the free Rpb4 did (Fig 5B) demonstrates that the cleavage between Rpb2 and Rpb4 occurred *in vivo*. If, however, this cleavage had occurred following cross-linking and cell lysis, we would have detected all the degradation products (detected by anti-Rpb4) also in the mRNA bound fraction.

Surprisingly, the Rpb2-Rpb4 fusion protein was also capable of binding mRNAs (Fig 5C, “Rpb2-4 fusion”), demonstrating that fusing Rpb4 to full length (and probably properly folded) Rpb2 does not abolish its capacity to bind mRNA. We have previously shown that binding of Rpb4 to mRNAs occurs only in the context of Pol II and Rpb7 [18,20]. In order to examine whether Rpb7 affects the binding of Rpb4 to mRNAs, we over-expressed a mutant form of Rpb7, which is defective in binding Rpb4 [29], in the *RPB2*-*RPB4* strain. Overexpressing the Rpb7-29 mutant decreased the capacity of the Rpb2-Rpb4 fusion protein to interact with mRNAs (Fig 5C). Thus, mRNA-binding capacity of the fusion proteins is affected by the Rpb7 mutation.

**Fig 6:**
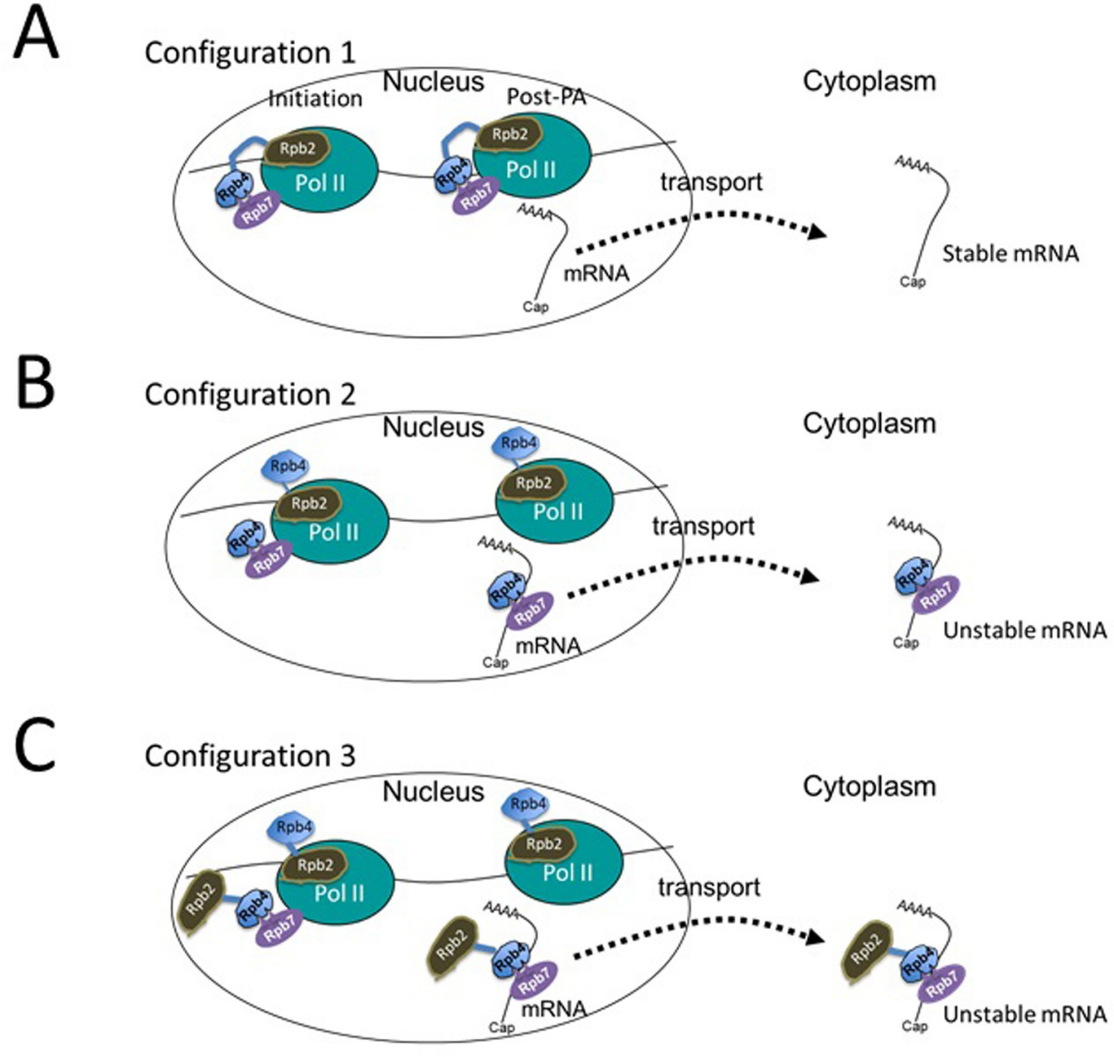
A proposed model: Different configurations of Pol II with different impact on post-transcriptional stage. The left Pol II complex represents initiating and elongating complex (for simplicity the nascent transcript is not shown), whereas the one at the right—the post-polyadenylation complex. The transcript represents mature one containing the 5’ cap and the 3’ poly(A) tail (represented as AAAA). Following transport, the same transcript is found in the cytoplasm (at the right). See also models in [18,20]. Rpb2-Rpb4 fusion is represented by its two moieties: Rpb2 (brawn), Rpb4 (blue), and the flexible linker between them is represented as a blue line (not drawn to scale). We propose the co-existence of three configurations of Pol II complex. “Configuration 1” that assembles the fusion protein as expected; Rpb2 and Rpb4 are placed in their natural positions and support normal transcription. The resulting transcript is exported without Rpb4 (and maybe without Rpb7) because Rpb4 cannot dissociate from Pol II. Since this transcript cannot bind Rpb4 after its dissociation from Pol II, the exported mRNA is relatively stable (Fig 2 and [15,20,21,40] and relatively poorly translated (Fig 4 and [18,19]). This configuration seems to be responsible for the uncoupling that we found between mRNA synthesis and decay. Note, however, that no proof was provided for the assumption that the fusion protein cannot dissociate from Pol II in complex with the transcript [15]. Such dissociation would require disassembly of the core Pol II, and we concur with Schulz et al. that this possibility is unlikely. In fact, the defective mRNA translatability and stability support this assumption. “Configuration 2” recruits a free Rpb4 (the cleavage product of Rpb2-Rpb4), which displaces the Rpb4 moiety of the fusion protein. The displaced Rpb4 is “idle”, and plays no active role in transcription. The Pol II-bound free Rpb4 functions like WT Rpb4, it binds mRNAs (Fig 5B) and polysome (Fig 4). Thus, this Pol II configuration permits normal post-transcriptional stages of the mRNA [18–21,23,40]. “Configuration 3” was inspired by the observations that the fusion protein exhibits features that characterize free Rpb4: it binds poly(A)+ mRNAs (Fig 5C), found in the cytoplasm [15] and a portion of it co-sediments with polysomes (Fig 4). This proposed configuration recruits two fusion proteins, one functions as Rpb2 (carrying an “idle” Rpb4) and the other functions as Rpb4 (carrying an “idle” Rpb2). The latter molecule binds Pol II only via binding of its Rpb4 moiety to Rpb7. Therefore, this molecule can dissociate from Pol II, bind the mRNA in an Rpb7-dependent manner (Fig 5C) and provides the function of Rpb4 in mRNA translation and degradation. It is quite possible that a mixture of Pol II configurations simultaneously transcribes genes. The ratio between the various configurations affects the apparent average translatability and stability of the mRNA. This model can provide plausible explanations for the effects that we observed upon overexpression of either (I) Rpb4/7 or (II) Rpb7-29. (I) Overexpression of Rpb4/7 leads to its increased binding with Pol II core complex, and out competes the Rpb4 moiety of the fusion protein for binding Pol II. As a result, configuration 2 increases on the expense of the others. Since only configuration 3 can support the co-transcriptional binding of the fusion protein with Pol II transcript, less fusion protein binds mRNA upon Rpb4/7 over-expression (Fig 5C). (II) Our understanding of the effect of Rpb7-29 is based on the following observations made previously. (1) The Rpb4/7 heterodimer binds Pol II via a small interface consists of the Rpb7 “tip” and a "pocket" in Pol II [1–3]. (2) Rpb7 can bind Pol II independently of Rpb4 [36], and its binding is increased upon Rpb7 overexpression [36]. (3) Rpb4, on the other hand, is recruited to Pol II via Rpb7 [1–3]. (4) The Rpb7-29 mutant form interacts poorly with Rpb4 [29]. Based on these observations, we propose that when the over-expressed Rpb7-29 is recruited to Pol II in lieu of Rpb7, free Rpb4 is poorly recruited to Pol II. Thus, the proportion of both configuration 2 and 3 decreases. Consistently, binding of the free Rpb4 and the fusion protein to mRNA is compromised upon over-expressing *rpb7-29* (Fig 5C, "Rpb2-4 fusion"). It is possible that configuration 1 is relatively less affected by the mutations in Rpb7-29 because, once the Rpb2 moiety of the fusion protein is recruited to Pol II, the Rpb4 moiety of the fusion protein is placed near Rpb7-29, increasing Rpb4 local concentration and push the interaction forward.

Lastly, we introduced a high-copy plasmid encoding both Rpb4 and Rpb7 ORFs into the *RPB2*-*RPB4* strain and evaluated its effect on the mRNA binding capacity of Rpb2-Rpb4 fusion protein. Expression of this high-copy plasmid leads to ~ 10 fold increase in the protein levels. Since both *RPB4* and *RPB7* ORFs, present in this plasmid, are controlled by their respective natural 5’ and 3’ non-coding regions, the natural ratio between Rpb4 and Rpb7 was maintained [22]. In these cells, Rpb4/7 is present in excess over Pol II core. Overexpressing Rpb4/7 led to a substantial reduction in the capacity of the Rpb2-Rpb4 fusion protein to bind mRNA (Fig 5C) suggesting that Rpb4/7 out competes the interaction of the fusion protein with mRNA. Note that over-expression of Rpb4/7 did not lead to a proportional increase in binding of Rpb4 with mRNAs (see Rpb4 in Fig 5C; compare the large difference in the input lanes and the smaller difference in the mRNA bound lanes). This is consistent with our finding that Rpb4 binds mRNA only in the context of Pol II [18,20]. That is, in these cells the extent of Rpb4-RNA interaction is proportional to the number of Pol II core components, not to the number Rpb4 and Rpb7 molecules (see Discussion and Fig 6 legend). Taken together, overexpressing *rpb7-29* or *RPB4/7* abrogates the capacity of the fusion protein to bind mRNA, suggesting that the fusion protein binds mRNAs in the context of Rpb7. This context-dependent binding also characterizes Rpb4 [18,20].

## Discussion

In all eukaryotic organisms that we have examined, whose sequence was determined, *RPB4* is encoded by a single distinct gene. Chimeric genes, such as *RPB2*-*RPB4*, represent unnatural entity that the cell has to cope with. Here we demonstrate that the product of this chimeric gene, the Rpb2-Rpb4 fusion protein, is partially deleterious, compromising mRNA translation and decay, cell proliferation and adaptability–e.g., response to stress. Specifically, while *RPB2-RPB4* recovers the transcriptional defect of *rpb4Δ* cells, it does not fully recover the defect of *rpb4Δ* cells in mRNA decay. The defect of *RPB2-RPB4* cells in mRNA decay is milder than that of *rpb4*Δ cells, yet the defects in DRs of the two strains are correlated (Fig 2A). Moreover, similar to *rpb4*Δ cells, *RPB2*-*RPB4* cells poorly cope with high temperature and ethanol. These effects are more severe in the presence of an Rpb7-29 mutant that is defective in binding Rpb4 (Fig 1B and 1C). In addition to these observations, here we report two mechanisms that help the cell to tolerate the fusion protein and to alleviate its deleterious effect. One mechanism involves cleavage of the fusion protein near the flexible region located between the two proteins [15], resulting in the release of a functional Rpb4 molecule. Mass spectrometric analysis of the free Rpb4 band revealed a peptide derived from Rpb4 N-terminus that contained also three glycin residues of this flexible region (results not shown). We have demonstrated that the cleavage site occurs *in vivo* and not following the cell lysis. First, free Rpb4 was detected following harsh protein extraction conditions that minimize any *in vitro* activity of incidental protease (S3 Fig). Second, free Rpb4, but not other Rpb2-Rpb4 degradation products, was cross-linked to mRNAs *in vivo* (see discussion to Fig 5B in the Results section). Moreover, the multiple cleavage sites or degradation intermediates that are observed in *RPB2-RPB4* cells, but not observed when WT Rpb2 or WT Rpb4 were analyzed (e.g., Figs 3 and S3), strongly suggest that the fusion protein is recognized as a substrate of a surveillance mechanism. However, free Rpb4 represents only ~10% of the total amount of the fusion protein, including the cleavage products. Nevertheless, because the fusion protein expression is 2.6-fold higher than Rpb4, it represents ~25% of Rpb4 level in WT cells. Considering our observation that cells that express only 54% of the normal Rbp4 level can proliferate like WT even under stress (S4 Fig), this amount of free Rpb4 seems effective. Yet, this level is not sufficient to restore WT phenotype, as was manifested by the slow proliferation rates, especially under adverse conditions, and by the poor capacity of these cells to translate and degrade mRNAs.

The second mechanism involves an unexpected feature of the Rpb2-Rpb4 fusion protein. We discovered that the fusion protein could bind mature poly(A)^+^ mRNAs (Fig 5C). Poly(A) addition occurs concomitantly with the dissociation of the nascent mRNA from Pol II [38]; hence, binding of the fusion protein to mature poly(A)+ mRNA indicates that either Rpb2-Rpb4 binds mRNA outside the context of the core Pol II, or Rpb2-Rpb4 dissociates from Pol II in complex with the RNA, much like Rpb4 does [18,20]. We found that overexpression of WT Rpb4/7, which binds mRNA only in the context of Pol II [18,20], decreases binding of Rpb2-Rpb4 to mRNA (Fig 5C). This finding argues against the first option. Consistently, a portion of the fusion protein is associated with polysomes (Fig 4) and is found in the cytoplasm [15]. It is worth emphasizing that the cytoplasmic volume is over one hundred fold larger than that of the nucleus; hence a small cytoplasmic signal represents a large proportion of fluorescent molecules in this compartment. Since the natural (endogenous) Rpb2 does not bind mRNAs (Fig 5A), this capacity is contributed by the Rpb4 moiety. We propose that binding of the fusion protein to mRNAs helps the cells to cope with the presence of the chimeric gene and that Rpb7-29 compromises this function (Fig 5C), thus slowing down cell proliferation in response to the environment (Fig 1B and 1C).

A plausible model that graphically illustrates the two mechanisms that alleviate the deleterious effect of the fusion protein is shown Fig 6 The basic principles that underlie this model posit that (i) Rpb4 binds Pol II via Rpb7 [1–3]; (ii) Rpb4 binds Pol II transcripts only co-transcriptionally—within the context of Pol II [18,20]. We propose that three different configurations of Pol II co-exist in *RPB2-RPB4* cells. We surmise that “configuration 1”, which is incompatible with a separation between Rpb4 and Pol II, is responsible for the uncoupling we observed between mRNA synthesis and decay. The two other configurations can maintain the coupling and alleviate the mRNA decay defect of *RPB2-RPB4* cells. We propose that the ratio between the three configurations determines the mRNA translatability and stability and that Rpb7-29 can impact this ratio. For example, as shown in Fig 1B and 1C, *RPB2-RPB4* cells are more sensitive to *rpb7-29* overexpression than WT cells. A plausible explanation for the adverse effect of Rpb7-29 on cell proliferation is through its differential effect on the various configurations (see Fig 6 legend).

Previously, investigators have confronted yeast cells with a new type of challenge, which they had not previously encountered. They have replaced the natural promoter of an essential gene, *HIS3*, with *GAL1* promoter and cultured these cells in glucose containing medium that represses the *GAL1* promoter. In response to the new challenge, the mutant cell reprogrammed gene expression. Strikingly, when the same experiment was repeated, different repertoire of mRNAs were changed–some exhibited reproducible changes and others exhibit nonreproducible changes, indicating that the cell is a plastic system whereby different solutions are possible [30–32]. Replacing the natural *RPB2* and *RPB4* with a chimeric gene seems to pause an analogous challenge. It is possible that there is more than one type of solutions and that those that we found are only examples of a number of potential solutions in the “arsenal”.

## Conclusions

The separation of Rpb4 from Pol II seems to be important for the normal coupling between mRNA synthesis and decay. Cells that lack *RPB2* and *RPB4* respond to Rpb2-Rpb4 and alleviate its adverse effect by at least two means. One involves cleaving the fusion protein in a manner that produces a free and functional Rpb4. In addition, the fusion protein is capable of binding mRNA via its Rpb4 moiety (see Fig 6). Under optimal growth conditions, these two features allow the cell to function similarly to WT, although not exactly as evidenced by their slower proliferation rate (Figs 1A and S1B), defective mRNA decay and translation (Figs 2A and 4). Importantly, defective coupling affects cell phenotype mainly under non-optimal conditions, suggesting that this coupling is required for proper responses to the environment.

## Materials and methods

### Yeast strains, plasmids and media

Yeast strains are depicted in S1 Table. To create *RPB2-RPB4*, we use the same shuffling assay and the same strain and the same plasmids created by Schulz et al [15] in which *RPB2*::*URA*3 plasmid was replaced by *RPB2-RPB4*::*LEU2* plasmid. Cells were grown in either YPD medium (2% Bacto Peptone, 1% yeast extract [Difco Laboratories], 2% dextrose), or synthetic complete (SC) medium lacking the appropriate amino acid as required (25). For all experiments, the inoculum was taken from cell cultures that were grown in log phase for at least seven generations. The plasmids are a generous gift of Schulz and Cramer [15].

### Cell harvest and grinding

Cells were harvested at 10^7^ cells/ml by filtration onto 0.45 μm pore size nitrocellulose filters (Whatman), using vacuum, scraped from the membrane and immediately submerged in liquid nitrogen. The frozen cell, was cryogenically pulverized for six cycles of 15 Hz for 30 sec on a Retsch MM301 mixer mill. Sample chambers were pre-chilled in liquid nitrogen and re-chilled between each cycle.

### Antibodies

Monoclonal anti-Rpb4 was purchased from Clonetec, anti Rpb1 from Santa Cruze and affinity purified anti-Rpb2 antibodies were a gift from A. Sentenac.

### Electrophoresis and Western blot analysis

Proteins (100–200 μg) were electrophoresed in 23 cm long 5–15% gradient polyacrylamide gel, and electro-transferred onto PVDF membrane and reacted with antibodies as described previously [18]. For S3 Fig, we used three extraction methods. Frozen cells (kept at -80°C) were cryogenically pulverized in the presence of liquid nitrogen, as detailed above. (i) For the standard method, 50 mg of grindate was dissolved in 500 μl of ice cold lysis buffer containing protease inhibitors (10 mM NaP buffer pH = 7.0; 1% NP-40; 100 mM KCl; 50 mM NaF; 0.1 mM Na_3_VO_4_; 10 mM beta-mercaptoethanol; 1x protease inhibitors cocktail (Rosch); 2 mM PMFS; 50 μg/ml TLCK; 2.5 mM Benzamidine). The sample was centrifuged at 20k g for 5 min at 4°C. The supernatant was supplemented with LSB (100 mM Tris HCl 6.8; 2% SDS; 5% α-mercaptoetanol; 4% glycerol; 0.1% bromophenol blue), boil for 5 min and loaded on a gel; (ii) For the TCA method, 50 mg grindate was dissolved in 400 μl of lysis buff. TCA was immediately added to a final concentration of 20%. Sample was incubated for 30 min on ice, centrifuged at 20k g for 5 min. The pellet was washed with cold acetone and briefly air dried followed by dissolving in LSB and boiling for ca 20 min until the pellet was mostly dissolved (not all cell derbies are dissolved). Sample was centrifuged for 30 sec and the supernatant was loaded on a gel. (iii) For the “48% Urea 7% SDS” method, 50 mg of the grindate was dissolved in 500 μl of (filtered) 100 mM Tris HCl 6.8, 48% Urea, 7% SDS, 10 mM EDTA and heated at 57°C for 10 min, centrifuged for 30 sec. and the supernatant was supplemented with bromophenol blue dye and load on a gel.

### Isolation of mRNA associated proteins

This procedure was performed as described previously [27], with some modifications. Briefly, cells grown in 1000 ml of SD media until 4x10^7^ cells/ml, were harvested by centrifugation, re-suspended with 25 ml of PBS containing 0.005% NP40, and transferred to a 150-mm plate disk. Plate disks were exposed to 1200 mJ/cm^2^ of 254 nm UV in a UV crosslinker (Stratagene) in three steps of 400 mJ/cm^2^ with two 2-min breaks on ice and gentle mixing. Cells were harvested by centrifugation at 4°C and re-suspended in 1.5 ml of lysis buffer (20 mM Tris pH 7.5, 0.5 M NaCl, 1 mM EDTA, 1 × protease inhibitor cocktail [Complete; Roche]). Frozen cells were broken by mixer mill as detailed above. Cell grindate was dissolved in 2 ml of lysis buffer (20mM Tris pH 7.5, 0.5M NaCl, 1mM EDTA, protease inhibitor cocktail [PIC-Roche]). The lysate was passed 3–5 times through a thin needle to break the chromatin and then clarified by a 10-min centrifugation at 14,000 rpm and 4°C, and 50 μl was kept as a control. Oligo (dT)_25_ cellulose beads (150 μl; New England BioLabs) were equilibrated with 500 μl of loading buffer (20 mM Tris–HCl pH 7.5, 0.5 M NaCl, 1 mM EDTA), spun, and mixed with the lysate. The mixture was incubated at room temperature for 60 min with gentle stirring and put in 10 ml column. The oligo (dT)_25_ cellulose beads were washed 4 times with of 10 ml of loading buffer containing 1% SDS and with 10 ml of low salt buffer (10 mM Tris–HCl pH 7.5, 0.1 M NaCl, 1 mM EDTA). Elution was performed by adding 250 μl of elution buffer (20 mM Tris–HCl pH 7.5; pre-warmed at 70°C) to the beads and incubating at room temperature for 5 min with gentle agitation. Elution was carried out twice and the two supernatants were mixed, lyophilized, and re-suspended in 35 μl of miliQ H_2_O. The mRNA content of the elute was quantified at 260 nm, using nano-drop. The RNA was then digested with RNase A + T1. Proteins from equal amounts of mRNA (before digestion) were analyzed by western blotting.

### Polysomal fractionation

Cells were allowed to proliferate in rich synthetic medium until mid-log phase (1x10^7^cells/ml). The cultures were supplemented with 100mg/ml cycloheximide (CHX) and immediately harvested and frozen by liquid nitrogen. Frozen cells were ground as indicated above. Each grindate was dissolved in 20 mM Tris HCl pH = 7.4; 140 mM KCl; 1.5 mM MgCl2; 0.5 mM DTT; 1% Triton X-100; 1 mg/ml heparin; 100 μM CHX; protease inhibitors cocktail and cleared by centrifugation at 20K g at 4°C for 10 min. One ml containing 2.5 mg of protein was loaded onto 10%–50% sucrose gradients, containing 20 mM Tris HCl pH = 7.4; 140 mM KCl; 5 mM MgCl2; 0.5 mM DTT; 0.05 mg/ml heparin; and centrifuged at 35K rpm at 4°C using a SW41 rotor, for 160 min. Fractions (0.75ml) were collected while scanning continuously at A254 using an ISCO gradient fraction collector. The fractions were supplemented with LSB (50 mM Tris-HCl pH 6.8, 2% SDS, 10% glycerol,1% β-mercaptoethanol, 12.5 mM EDTA, 0.02% bromophenol blue), loaded on 23 cm long SDS PAGE gels and analyze by western blot analysis.

### Proliferation competition by co-culturing

Exponentially proliferating cells were mixed as follows: Culture A contained equal number of WT cells carrying p*RPB2*::*URA3* (yMC797) and WT cells carrying p*RPB2*::*LEU2* (yMC798). This culture of two identical WT strains, which differ only in their selectable marker, was used as a control culture and for normalization. Culture B contained equal number of WT cells carrying p*RPB2*::*URA3* (yMC797) and *RPB2-RPB4 cells* carrying p*RPB2-RPB4*::*LEU2* (yMC871). Cells were allowed to proliferate on rich medium with no selection, because the plasmids carry essential genes and could not be lost. Rich medium (YPD) was used to permit the best possible conditions. The experiment begun by plating equal amount of cells on a selective (SC lacking uracile) plates (at least two plates), permitting growth of the *URA3*+ and a second set of plates (SC lacking uracile) permitting growth of *LEU2+ cells*. The cultures were diluted 1000 fold and shaked at 3°C for 10 generations (until the culture reached the same cell density as before the dilution), followed by plating as above. The ratio between *LEU2* and *URA3* expressing colonies of culture B was determined; it was normalized to that of culture A (that normalized the impact of the selectable markers on cell proliferation). This process was repeated several times.

## Supporting information

S1 Table Yeast strains. (DOCX)

S1 Fig (A) Level of Tet-off-RPB2 product as a function of time after doxycycline addition. (B). RPB2-RPB4 cells proliferate more slowly than WT cells in a co-culture.
(PDF)

S2 Fig [Lack of] Correlation between mRNA decay rates in *RPB2-RPB4* cells against various mutant strains, each carries a deletion in a certain gene encoding mRNA decay factor. (PDF)

S3 Fig Detection of free Rpb4 using different methods of protein extraction. (PDF)

S4 Fig Cells that express ~ one half of the normal level of Rpb4 can proliferate like WT at high temperature. (PDF)
