# Supplementary material for: Dissociation of Rpb4 from RNA polymerase II is important for yeast functionality
Source: PLoS One. 2018 Oct 25;13(10):e0206161. doi: 10.1371/journal.pone.0206161 (PMC6201915; doi:10.1371/journal.pone.0206161)
Supplement: S1 Table — (DOCX) [file pone.0206161.s001.docx]

**Supporting information**

**S1 Table.** **Yeast strains**

| Strain | Description | Genotype | Origin |
| --- | --- | --- | --- |
| yMC797 | "Shuffle strain" | MATa; *his3Δ1; leu2Δ0;*  *met15Δ0; ura3Δ0;*  *rpb2*Δ::KanMX6; p*RS316-RPB2* | Schulz et al., 2014 (15) |
| yMC798 | "WT" | MATa; *his3Δ1*; *leu2Δ0*;  *met15Δ0 ; ura3Δ0*  *rpb2*Δ::KanMX6; p*RS315-RPB2* | Schulz et al., 2014 (15) |
| yMC799 | “Rpb2-Rpb4 fusion" | MATa; *his3Δ1; leu2Δ0; met15Δ0 ;LYS2;*  *ura3Δ0; rpb2::KanMX6; rpb4Δ::*clonNAT; p*RS315-*  *RPB2-RPB4* | Schulz et al., 2014 (15) |
| yMC800 | “∆*rpb4*” | MATa; *his3Δ1; leu2Δ0;*  *met15Δ0; ura3Δ0;*  *rpb2Δ::*KanMX6*; rpb4Δ::*clonNAT; p*RS315-RPB2* | Schulz et al., 2014 (15) |
| yMC871 | Recreation of yMC799 | MATa; *his3Δ1; leu2Δ0; met15Δ0; LYS2;*  *ura3Δ0; rpb2::*KanMX6; *rpb4Δ::*clonNAT; *pRS315-*  *RPB2-RPB4* | This work. |
| yMC877 | yMC871 carrying *RPB3*-TAP::*URA3* | MATa; *his3Δ1; leu2Δ0; met15Δ0 ;LYS2;*  *ura3Δ0; rpb2*::KanMX6*; rpb4Δ::*clonNAT; *RPB3-TAP::URA3;* p*RS315-*  *RPB2-RPB4*; | This work |
| yMC884 | Tet-off-*RPB2* | MATa; *his3-1; leu2-0; met15-0; RPB2*p::kanR-tet07-TATA *URA3*::CMV-tTA | Tet-off library (Euroscarf) |
| yMC886 | yMC884 carrying *rpb4Δ::*clonNAT | MATa; *his3-1 leu2-0; met15-0;* RPB2p::kanR-tet07-TATA URA3::CMV-tTA; *rpb4∆*::cloNAT | This work |
| yMC921 | Y871+ pMC279 (high copy plasmid carrying *RPB4* and *RPB7)* | MATa; *his3Δ1; leu2Δ0; met15Δ0 ;LYS2;*  *ura3Δ0; rpb2::KanMX6; rpb4Δ::*clonNAT; p*RS315-*  *RPB2-RPB4*; p*RS426-RPB4/7* | This work |
| yMC923 | Y798+ pMC742 (high copy plasmid carrying *rpb7-29).* | MATa; *his3Δ1*;  *leu2Δ0*; *met15Δ0 ; ura3Δ0*  *rpb2*Δ::KanMX6; p*RS315-RPB2;* p*CM190*-*rpb7-29* | This work |
| yMC924 | Y871+pMC742 (high copy plasmid carrying *rpb7-29).* | MATa; *his3Δ1; leu2Δ0; met15Δ0 ;LYS2;*  *ura3Δ0; rpb2::*KanMX6*; rpb4Δ::*clonNAT; p*RS315-RPB2-RPB4;* p*CM190*-*rpb7-29* (high copy) | This work |
| yMC958 | *rpb4Δ* + p*GAL1*p-*RPB4* | MATa, *rpb4::HIS3*; *lys801*(amber); *leu2-3,2-112; trp1-1* (amber); *ura3-52*; his3Δ200.+ pYcp50-*GAL1*p-*RPB4* | This work |
| yLD41 | yMC884+ pMC645 (p*RPB2*) | MATa; *his3-1; leu2-0; met15-0; RPB2*p::kanR-tet07-TATA *URA3*::CMV-tTA + p*RS315-RPB2* | This work |
| yLD42 | yMC884 + pRS315 | MATa; *his3-1; leu2-0; met15-0; RPB2*p::kanR-tet07-TATA *URA3*::CMV-tTA + pRS315 | This work |
| yLD45 | yMC866 + p*RPB2*-*RPB4* | MATa; *his3-1; leu2-0; met15-0; RPB2*p::kanR-tet07-TATA *URA3*::CMV-tTA; *rpb4*Δ::clonNAT; p*RS315-RPB2-RPB4* | This work |
|  |  |  |  |
|  |  |  |  |

**Supporting Information Figure Legends**

S1 Fig **(A)** **Level of Tet-off-RPB2 product as a function of time after doxycycline addition**. yLD45 cells were treated with 10 µg/ml doxycycline. Cells were harvested at the indicated time points, their proteins were extracted and equal amount of protein was loaded per lane and analyzed by western blot, using anti-Rpb2 Abs. The non-specific band (marked by asterisk) serves as a loading control. Note that the level of Rpb2-4 protein in yLD45 is higher than Rpb2 protein in the WT strain (yMC798). **(B)**. ***RPB2-RPB4* cells proliferate more slowly than WT cells in a co-culture**. Competitive proliferation assay was performed by co-culturing yMC979 (expressing *URA3*) and yMC871 (expressing *LEU2*) (Culture B), and, as a control, co-culturing of two WT strains, yMC979 (expressing URA3) and yMC978 (expressing LEU2) (Culture A). Competition assay was performed as detailed in Materials and methods. The ratio between *LEU2* and *URA3* expressing colonies of culture B was normalized to that of culture A (that normalized the impact of the selectable markers on cell proliferation). Each cycle represents a competition feature after 10 generations (see Materials and methods). The histogram shows an average of two biological replicates. Error bar represents standard deviation.

**S2 Fig.** [Lack of] **Correlation between mRNA decay rates in *RPB2-RPB4* cells against various mutant strains, each carries a deletion in a certain gene encoding mRNA decay factor.** mRNA decay rates (DRs) data for *rpb4*∆ and for *RPB2-RPB4* strains were taken from Schulz et al. [[15](#_ENREF_15)]; DR data of the other strains were taken from [[39](#_ENREF_39)]. Fold changes in DRs for a given mutant cells (log_2_ folds of mutant/WT, y axis), against fold changes in DRs for *RPB2-RPB4* cells (log_2_ folds of *RPB2-RPB4*/WT, x axis), was performed as described in Fig 2. The deleted gene is indicated in the y-axis title. All strains are derivative of BY4741 [[15](#_ENREF_15),[39](#_ENREF_39)]. Related to Figure 2.

S3 Fig. **Detection of free Rpb4 using different methods of protein extraction*.*** Live cells were frozen in liquid nitrogen and ground in the presence of liquid nitrogen as described in Materials and methods. Three methods of dissolving the gradates and further processing the samples were used, as indicated above the autoradiograms (see Materials and methods). Cell lysates of the indicated strains were loaded on polyacrylamide gel; each lysate was loaded in 3 lanes, of 3-fold dilutions (except for the Urea/SDS lysates that were diluted only once). Two replicates were thus loaded and analyzed by Western blotting assay; one membrane was reacted with either anti-Rpb4 Abs and the second with a combination of anti-Dhh1 + anti-Tif35. Ponsau S stain of the upper membranes is also shown. The pre-stained size marker (BioRad) is also shown (designated ""M").

**S4 Fig. Cells that express ~ one half of the normal level of Rpb4 can proliferate like WT at high temperature. (A)** Cells carrying the indicated construct were allowed to proliferate either in a medium containing 2% galactose as the major carbon source (conditions that activate *GAL1* promoter), or in a medium containing 2% glucose as the major carbon source (conditions that repress *GAL1* promoter), as indicated (Gal – galactose; Glu – glucose). Cell lysates of the indicated strains were loaded on polyacrylamide gel in two replicates, each replicate was analyzed by Western blotting assay; one was reacted with either anti-Rpb4 (left panel), and the second with a combination of anti-Dhh1 + anti-Tif35. **(B)** Quantification of the results shown in A. Rpb4 level (normalized to the signals of Tif35 and Dhh1) was quantified relative to WT Rpb4, which was defined arbitrarily as 100%. Standard deviation is shown (n=3). **(C)** Spot test of 5-fold serial dilution of the indicated strains grown on selective plates containing 2% glucose as the main carbon source. The first spot of each strain was of 5000 cells. The plates were incubated at either 24°C or 37°C, as indicated, for 3 days.
