# Supplementary figures and images for: Dissociation of Rpb4 from RNA polymerase II is important for yeast functionality
Source: PLoS One. 2018 Oct 25;13(10):e0206161. doi: 10.1371/journal.pone.0206161 (PMC6201915; doi:10.1371/journal.pone.0206161)

# A

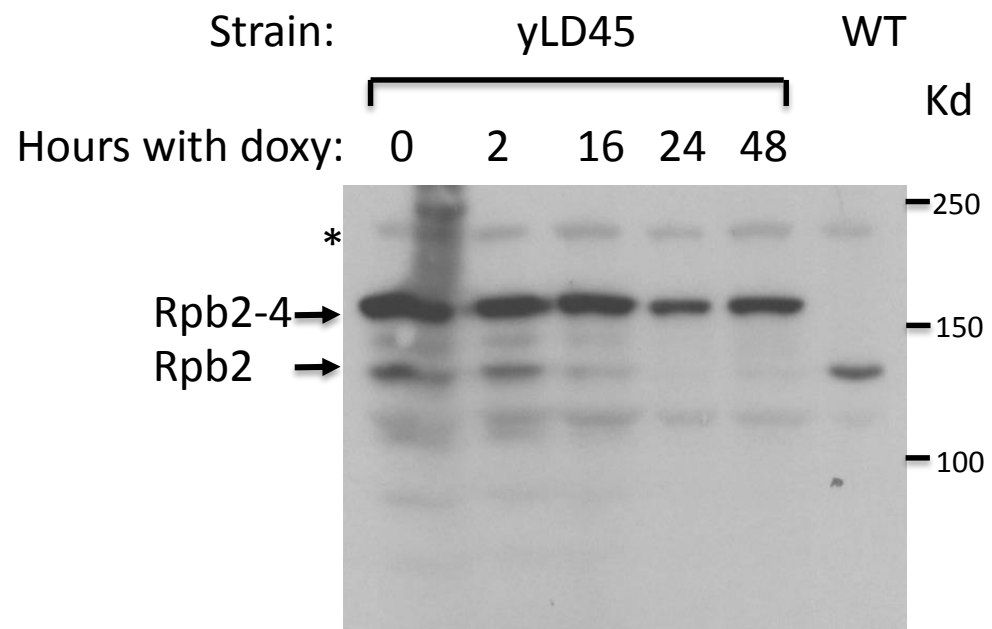

# B

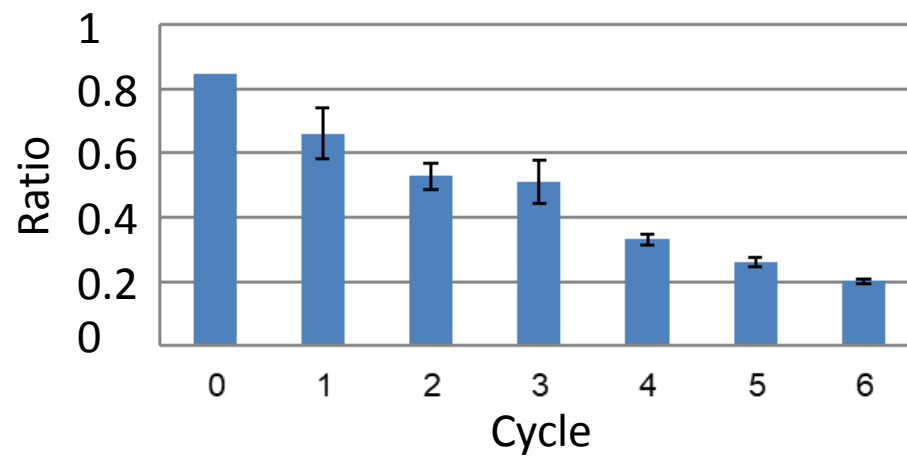

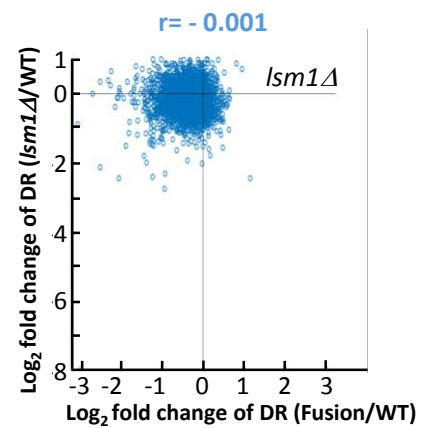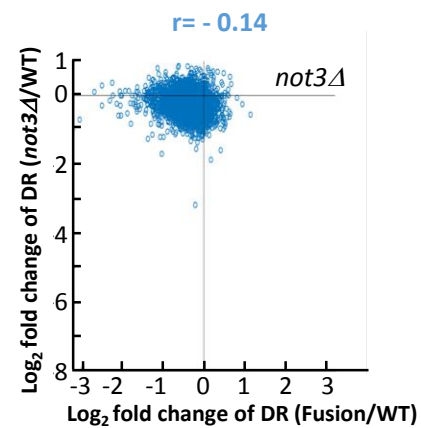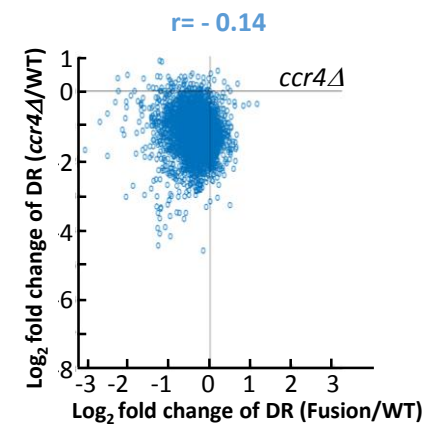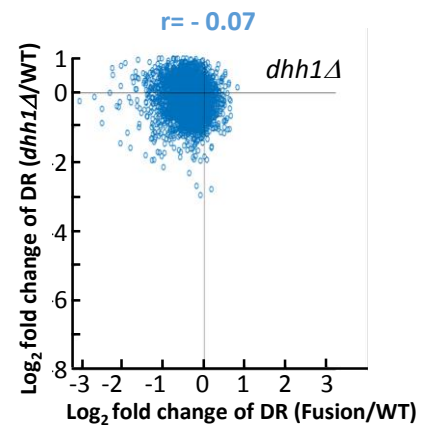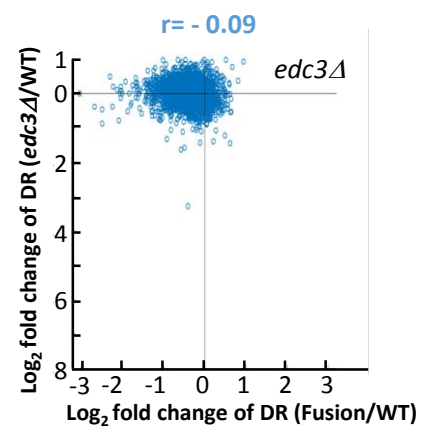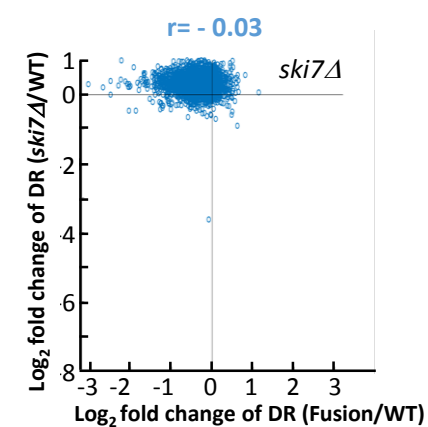

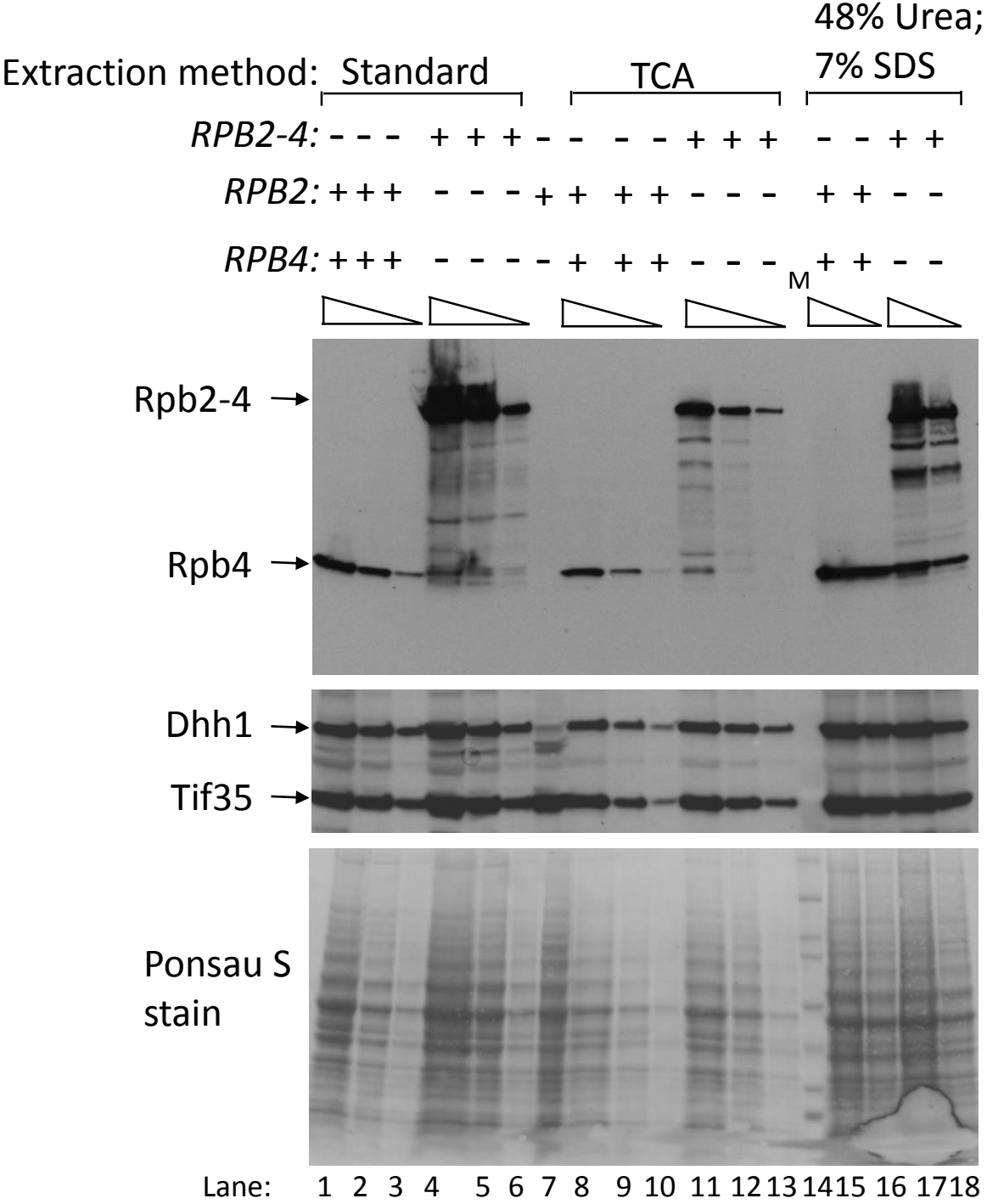

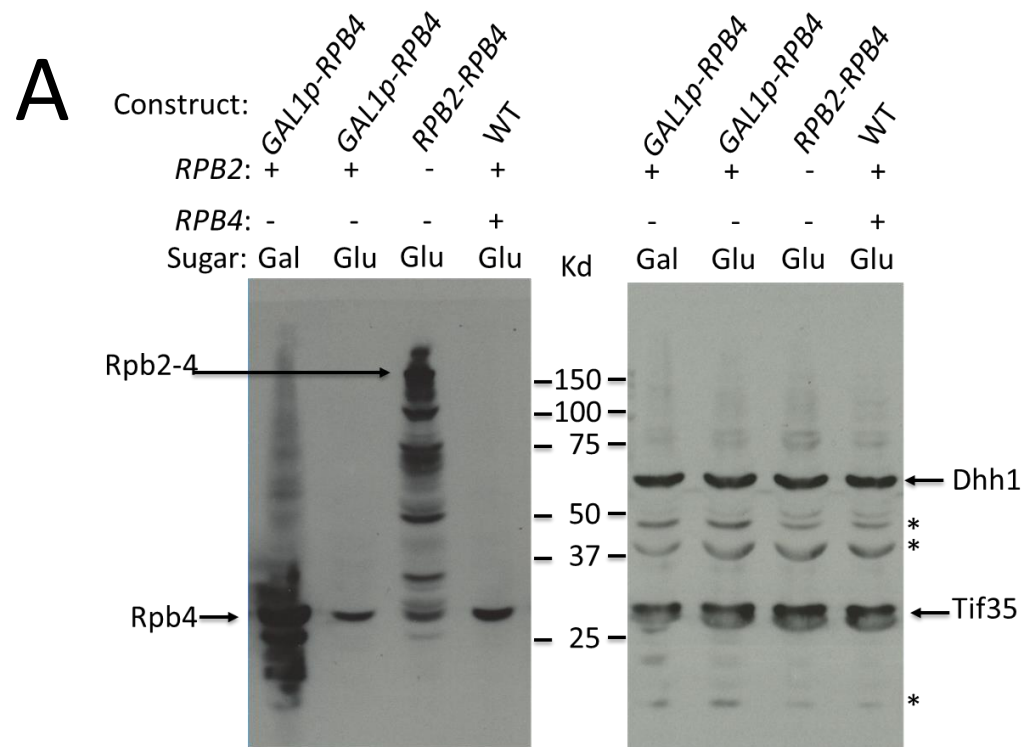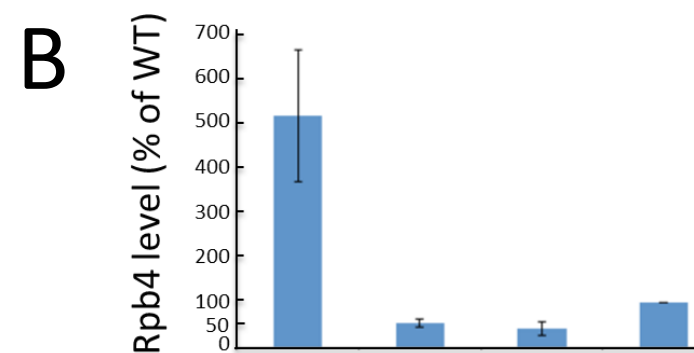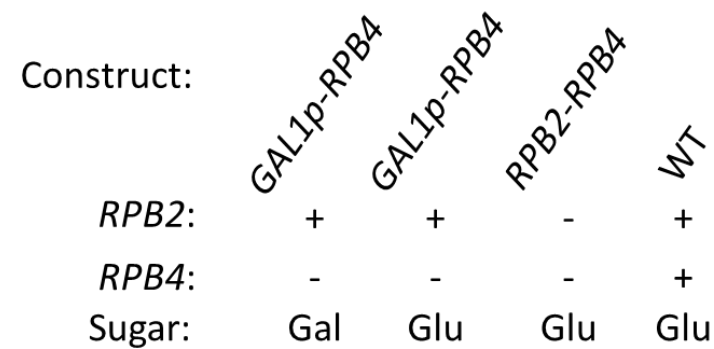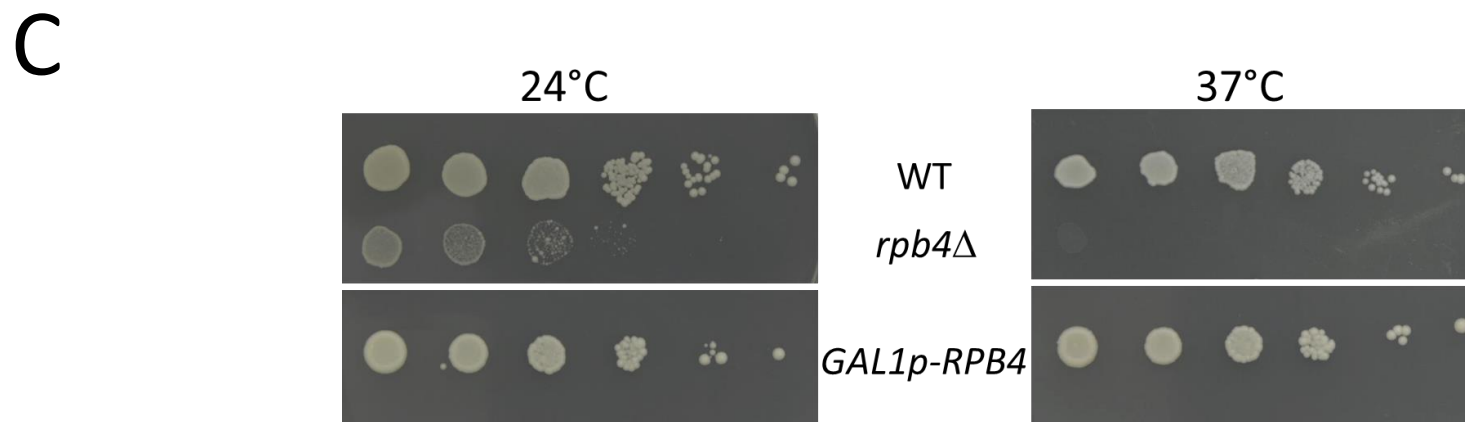

Supplement: S1 Fig — (A) Level of Tet-off-RPB2 product as a function of time after doxycycline addition. (B). RPB2-RPB4 cells proliferate more slowly than WT cells in a co-culture. (PDF) [file pone.0206161.s002.pdf]

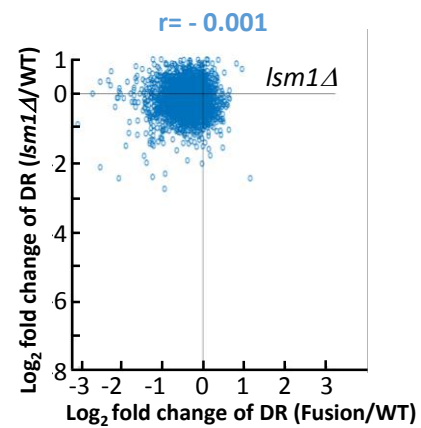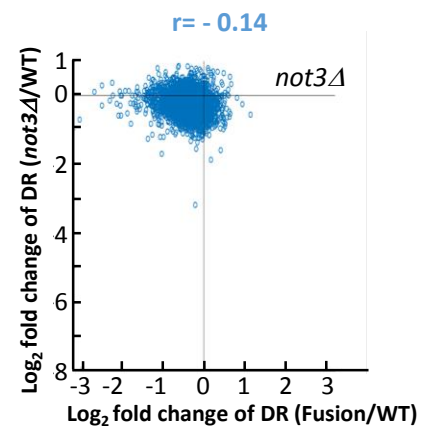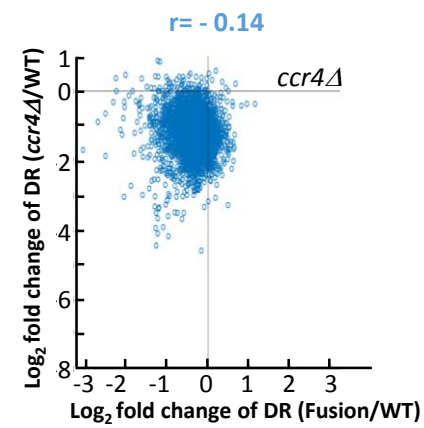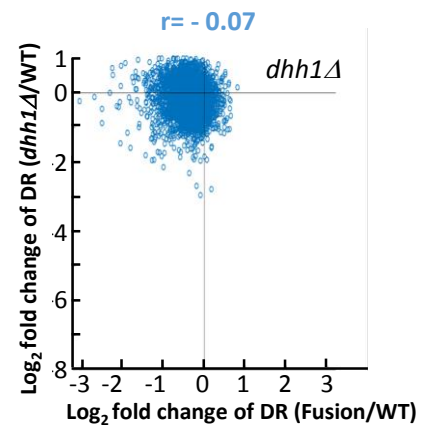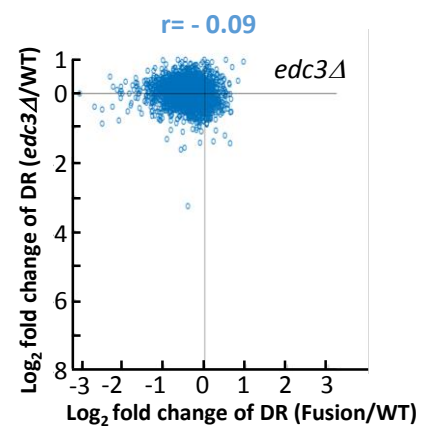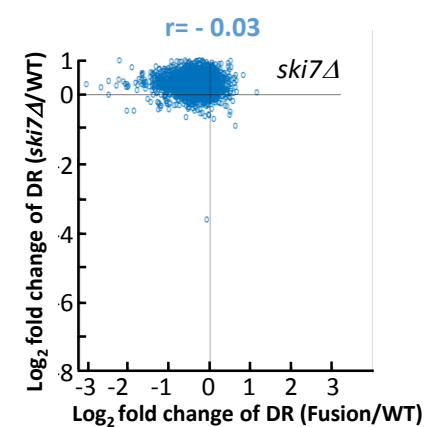

Supplement: S2 Fig — (PDF) [file pone.0206161.s003.pdf]

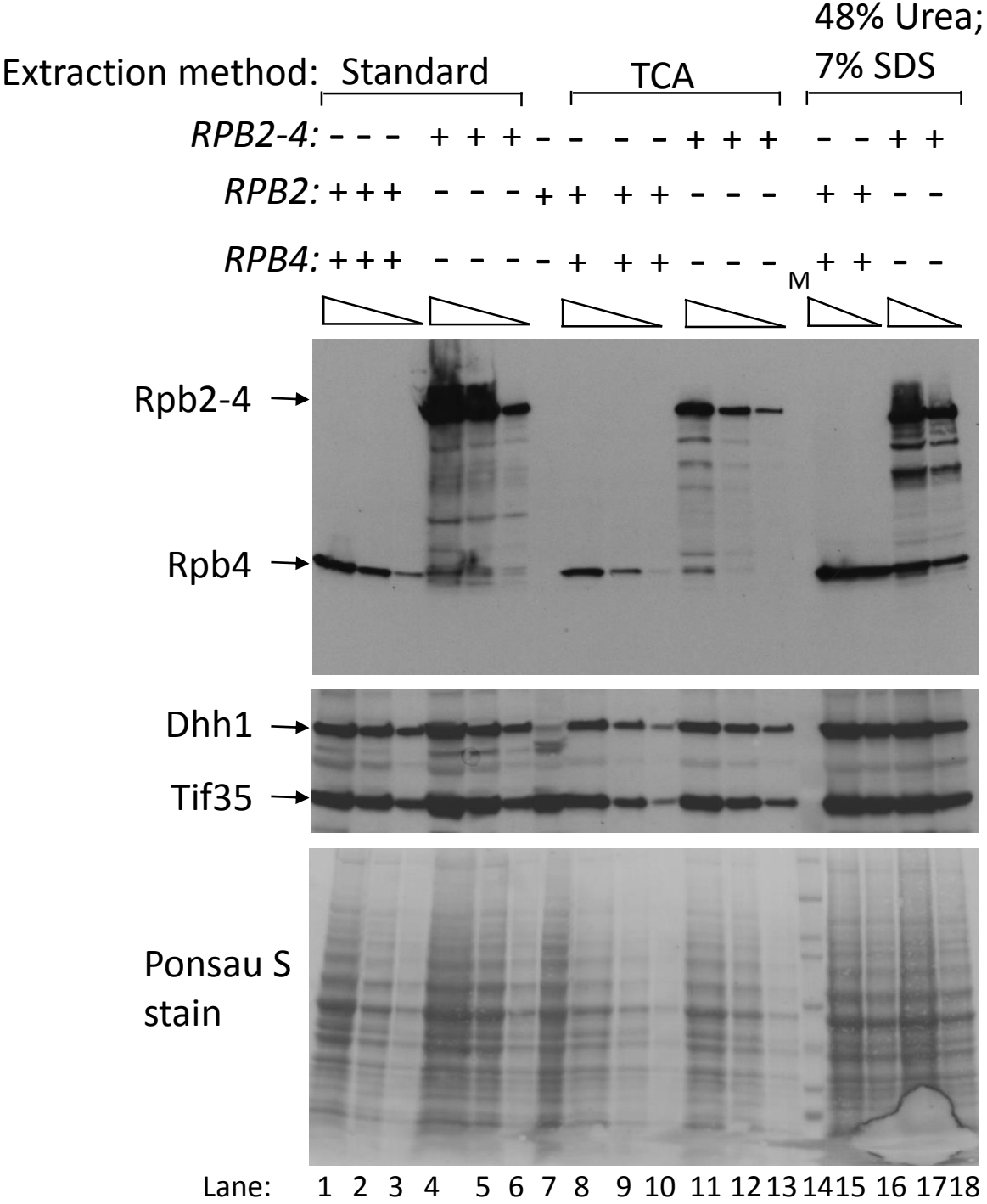

Supplement: S3 Fig — (PDF) [file pone.0206161.s004.pdf]

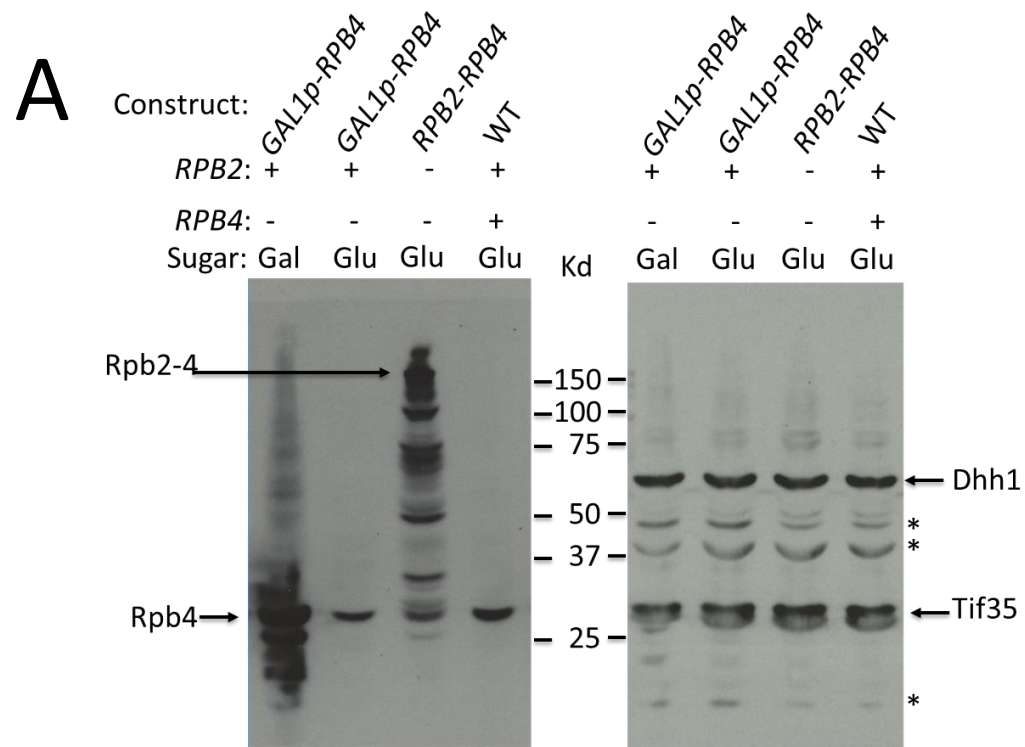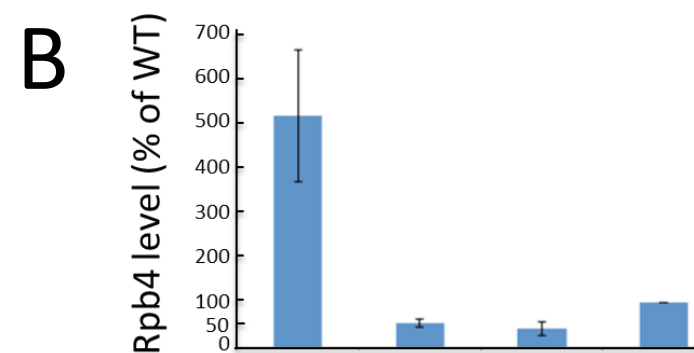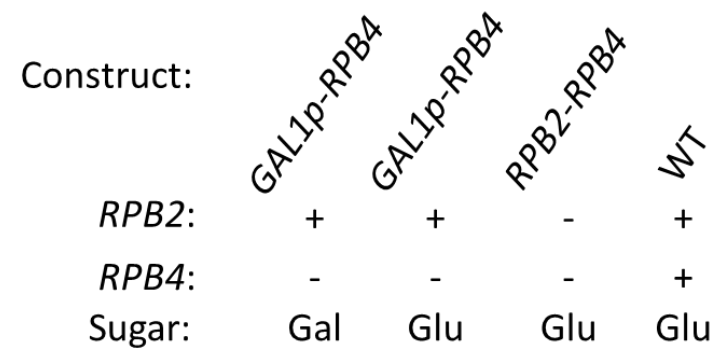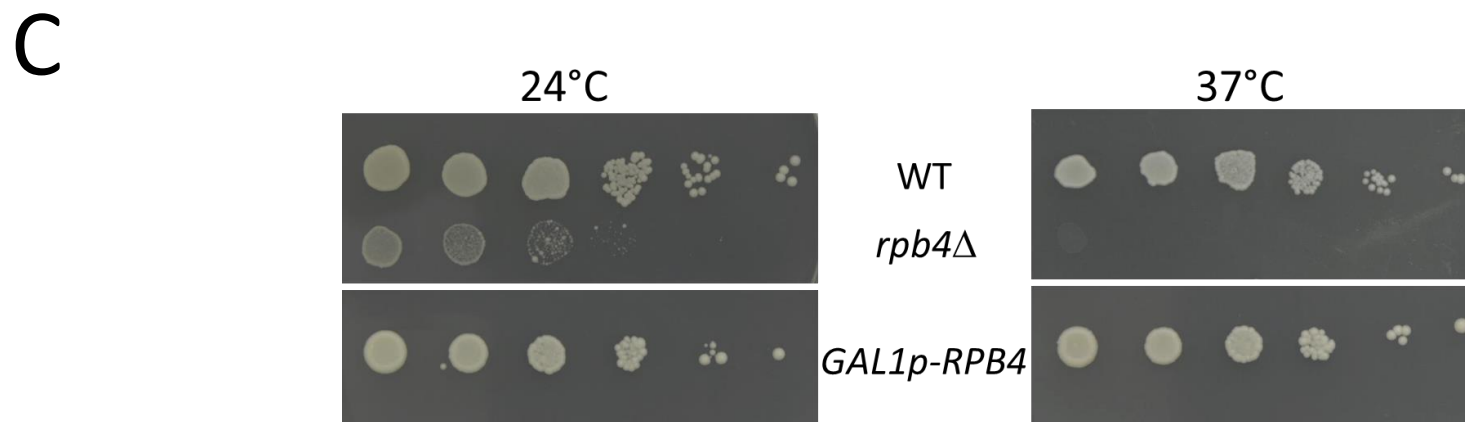

Supplement: S4 Fig — (PDF) [file pone.0206161.s005.pdf]
